# Diets maintained in a changing world: Does land‐use intensification alter wild bee communities by selecting for flexible generalists?

**DOI:** 10.1002/ece3.8919

**Published:** 2022-05-15

**Authors:** Birte Peters, Alexander Keller, Sara Diana Leonhardt

**Affiliations:** ^1^ Department for Animal Ecology and Tropical Biology University of Würzburg Biocenter Würzburg Germany; ^2^ Department of Bioinformatics University of Würzburg Biocenter Würzburg Germany; ^3^ Center for Computational and Theoretical Biology University of Würzburg Würzburg Germany; ^4^ Cellular and Organismic Networks Faculty of Biology Ludwig‐Maximilians‐Universität Munich Planegg‐Martinsried Germany; ^5^ 9184 Department of Life Science Systems Technical University of Munich Freising Germany

**Keywords:** Bee decline, biodiversity exploratories, foraging, metabarcoding, pollen nutrients, solitary bees

## Abstract

Biodiversity loss, as often found in intensively managed agricultural landscapes, correlates with reduced ecosystem functioning, for example, pollination by insects, and with altered plant composition, diversity, and abundance. But how does this change in floral resource diversity and composition relate to occurrence and resource use patterns of trap‐nesting solitary bees? To better understand the impact of land‐use intensification on communities of trap‐nesting solitary bees in managed grasslands, we investigated their pollen foraging, reproductive fitness, and the nutritional quality of larval food along a land‐use intensity gradient in Germany. We found bee species diversity to decrease with increasing land‐use intensity irrespective of region‐specific community compositions and interaction networks. Land use also strongly affected the diversity and composition of pollen collected by bees. Lack of suitable pollen sources likely explains the absence of several bee species at sites of high land‐use intensity. The only species present throughout, *Osmia bicornis* (red mason bee), foraged on largely different pollen sources across sites. In doing so, it maintained a relatively stable, albeit variable nutritional quality of larval diets (i.e., protein to lipid (P:L) ratio). The observed changes in bee–plant pollen interaction patterns indicate that only the flexible generalists, such as *O*. *bicornis*, may be able to compensate the strong alterations in floral resource landscapes and to obtain food of sufficient quality through readily shifting to alternative plant sources. In contrast, other, less flexible, bee species disappear.

## Graphical abstract

Our study provides new insight into the importance of land‐use induced changes for pollinator communities. It combines extensive field observations, pollen metabarcoding and comprehensive chemical‐nutritional analyses. We show that solitary bee species diversity decreased with increasing land‐use intensity and decreasing floral diversity with one bee species (*Osmia bicornis*) occurring over the entire gradient. Our detailed analyses reveal that this species is characterized by extreme flexibility in larval diets and nutrient intake ratios. We discuss the hypothesis that intensified land‐use, as found in modern landscapes, acts as a filter for flexible generalists, but excludes less flexible species.

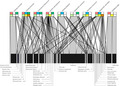

## 1 BACKGROUND

The current global insect decline threatens the resilience of insect‐associated ecosystem functions (Garibaldi et al., 2013; Potts et al., 2010; Seibold et al., 2019; van Klink et al., 2020; Wagner, 2020; Wagner et al., 2021). For example, the loss of insect pollinators puts the pollination of pollinator‐dependent wild and crop plant species at risk (Aizen et al., 2009; Klein et al., 2007; Kremen et al., 2002). Insect pollinators, such as wild bees, are endangered by multiple factors, of which agricultural intensification is one of the most severe (Ghazoul, 2013; Goulson et al., 2015; Quintero et al., 2010; Raven & Wagner, 2021; Steffan‐Dewenter & Tscharntke, 1999; Stout & Morales, 2009; Winfree et al., 2009). Intensified land use is typically associated with strongly reduced floral diversity and abundance and altered plant community composition (Blüthgen & Klein, 2011; Kaluza et al., 2016, 2018; Newbold et al., 2015; Requier & Leonhardt, 2020; Weiner et al., 2011). At the extreme, this can lead to largely homogenous landscapes with similarly homogenous plant and animal communities of very low diversity (Dormann et al., 2007). Here, monocultures of mass‐flowering crops, such as oilseed rapes, can—where present—provide timely restricted large amounts of food for bees, but do typically not compensate for the lack of flowers and thus food resources before and after mass‐flowering (Holzschuh et al., 2016; Riedinger et al., 2015). However, negative effects of such intensively managed homogenous landscapes on, for example, trap‐nesting bees appear to be (at least partly) mitigated by additional floral resources as, for example, provided by semi‐natural habitats in the surrounding landscape (Dainese et al., 2018; Persson et al., 2018) and even at smaller experimental scales (Ebeling et al., 2012). Notably, both positive effects of additional floral resources and negative effects of land use appear to strongly depend on the scale considered and the bee species studied (Dainese et al., 2018; Fabian et al., 2014; Hopfenmüller et al., 2020; Steckel et al., 2014; Weiner et al., 2011).

In general, changes in the floral resource landscape can reduce the quantity, taxonomic diversity (Carvell et al., 2006; Potts et al., 2010), and nutritional quality of food with still poorly understood effects on bee populations (Vaudo et al., 2015). In fact, larvae of both specialist and generalist bee species show reduced performance or fail to develop on inappropriate pollen diets (Dharampal et al., 2020; Eckhardt et al., 2014; Moerman et al., 2017; Sedivy et al., 2011), highlighting the role of diverse pollen sources to support diverse bee communities (Waser & Ollerton, 2006). As bees are central‐place foragers and are therefore restricted in their foraging ranges (Greenleaf et al., 2007), the composition of floral communities surrounding nesting sites strongly determines whether or not bees can access the pollen sources required to successfully raise offspring (Wilson et al., 2020). How precisely changes in the resource landscape as a consequence of, for example, intensified land use affect the taxonomic and chemical composition of pollen used by bees to provision their offspring has so far been little investigated (Filipiak & Weiner, 2017; Watrous et al., 2019). Recent advances in molecular and analytical methods, such as pollen DNA metabarcoding or new analytical protocols to analyze pollen nutrients (Danner et al., 2017; Sickel et al., 2015; Vanderplanck et al., 2011), provide novel tools to address these knowledge gaps. Unraveling the relationship between land‐use‐induced changes in plant community composition and bee species‐specific patterns in taxonomic and chemical resource use is, in turn, essential for understanding the effect of land use on bee pollinator population dynamics and thus declines.

In this study, we investigated how land‐use intensification affects the diversity and composition of trap nesting solitary bee species communities as well as the spectrum of pollen collected for larval provision in managed grasslands across three bioregions in Germany. We expected to find more bee species and a more diverse composition of plant species in larval pollen provisions at field sites with comparatively low agricultural intensification. We also expected the complexity of bee–plant interaction networks to decrease with increasing land‐use intensity as a consequence of impoverished bee and plant communities. We found only one bee species to be present across all three investigated bioregions and along the entire land‐use intensity gradient, *Osmia bicornis*. For this species, we additionally assessed fitness relevant factors, that is, nutritional quality of pollen provisions and numbers of brood cells per nest, to better understand how this species managed to thrive across the entire land‐use gradient.

## 2 MATERIAL AND METHODS

### 2.1 Sampling area and study design

The study was carried out from March to October in 2017 and 2018 on 27 grassland plots in three geographically separated regions as part of the German Biodiversity Exploratories (Figure SM1). In each of the three regions, nine experimental grassland plots were selected to cover different intensities and combinations of land‐use management, including meadows, pastures mowed or grazed by livestock as well as fertilized and unfertilized plots (Blüthgen et al., 2012) (Table SM1). Each experimental plot covers and area of 50 × 50 m, but grasslands and respective land‐use span beyond this area (Fischer et al., 2010).

In early spring 2017, four artificial perpendicular solitary bee trap nests with hollow reed internodes (Staab et al., 2018), pointing in every compass direction, were installed at the fence of a weather station located on each of the 27 plots (Figure SM1). We collected samples (i.e., occupied reed internodes) five times in 2017 and three times in 2018.

In the laboratory, reed cane internodes were opened lengthwise. Bee species were identified by reed nest closures and bee morphology according to (Amiet et al., 2017). Brood cells per species and site were counted as a proxy measure for reproductive fitness. To investigate how land‐use and subsequent changes in plant species diversity and composition affected the taxonomic composition of larval pollen provisions, we collected pollen provisions from nests of bee species which were found at at least three sites across bioregions. Per reed, a maximum of 3–5 pollen provision samples were collected with sterile forceps, pooled, and weighed to measure the total amount of wet pollen provision per reed. We used left‐over pollen provisions in reed cells with well‐developed larvae and pupae. Thus, we made sure to only sample minute amounts of pollen per cell as to not severely impair larval development.

In total, 150 pooled pollen provision samples of nine bee species were collected: 90 samples for *Osmia bicornis*, 15 for *O*. *caerulescens*, 7 for *O*. *cornuta*, 3 for *O*. *leaiana*, 11 for *Megachile rotundata*, 7 for *M*. *versicolor*, 7 for *Chelostoma florisomne*, 7 for *Heriades truncorum*, and 3 for *Hylaeus* spp. We only used pooled samples with at least 25 mg larval pollen provision to standardize input volumes for pollen metabarcoding (~5–7 mg) and for nutritional analyses (~10–20 mg).

### 2.2 Metabarcoding

Genomic DNA isolation was conducted with the Macherey‐Nagel Nucleospin (Düren, Germany) kits for food and according to the supplementary protocol for pollen (Keller et al., 2014). We followed the dual‐indexing strategy based on (Sickel et al., 2015) in order to generate a pooled amplicon library based on the ITS2 rDNA region used for pollen metabarcoding for the Illumina platform (Illumina, 2017) (see SM **Analytical details** for Metabarcoding and bioinformatics workflow).

### 2.3 Nutritional analyses

To assess how land‐use‐induced differences in the diversity and composition of plant species used for pollen collection by *O*. *bicornis* affected the nutritional composition of larval pollen provisions, we analyzed the composition of both amino acids and fatty acids in pollen provisions. The composition of amino acids (free and protein‐bound pooled) of larval pollen provisions of *O*. *bicornis* was analyzed by ion exchange chromatography (IEC: Biochrom 20 *plus* amino acid analyzer) following (Leonhardt & Blüthgen, 2011) (see SM **Analytical details**). Total protein content was calculated as the sum of all amino acids. The composition of fatty acids in larval provisions was analyzed by gas chromatography and mass spectrometry (GCMS: 5975C intert XL MSD, Agilent Technologies) following Brückner et al. (2017) with procedures adapted as detailed in SM (**Analytical details**). Total fat content was calculated as the sum of all fatty acids.

### 2.4 Statistical analysis

Data were analyzed in *R* 3.5.2. (R core, 2017) using the packages *phyloseq v1*.*22*.*3* (McMurdie & Holmes, 2013), *vegan v2*.*5*–*2* (Oksanen et al., 2013, *ggplot2 v3*.*0*.*0* (Wickham, 2009), *reshape2 v1*.*4*.*3* (Wickham, 2007), *bipartite v2*.*11* (Dormann, Fruend & Gruber, 2009), *lme4 v1*.*1*–*21* (Bates et al, 2015), *lmerTest v3*.*1*–*3* (Kuznetsova et al., 2017), *MASS v7*.*3*–*53*.*1* (Venables & Ripley, 2002), *multcomp v1*.*4*–*10* (Hothorn et al., 2008), *MuMIn v1*.*43*.*6* (Barton,^2019^), *psych v1*.*8*.*12* (Revelle, 2018), and *corrplot v0*.*84* (Wei & Simko, 2017).

We investigated how several explanatory variables related to land use affected our response variables. Explanatory variables were land‐use intensity (LUI), an index used as standard measure in the Biodiversity Exploratories framework (Blüthgen et al., 2012), its components grazing, mowing, and fertilization, as well as flowering plant species richness (dataset provided by Biodiversity Exploratories). Flowering plant species richness was included as it correlates with LUI and the number of flower visitor‐plant interactions (Weiner et al., 2011, 2014). Flowering plant species was assessed at the same plots as used in our study (also see details in **Data availability**). We composed a rank correlation matrix (*psych package*) to determine significant correlations between all our response and explanatory variables (Supplemental material, Table SM2).

We always conducted two separate generalized linear mixed‐effect model analyses (GLMMs, *lme4 package*) to assess if individual (non‐correlating) components of LUI or LUI itself better explained our results. Thus, one GLMM comprised flowering plant species richness and land‐use intensity (LUI) as fixed factors (Supplemental material, Table SM1). The other model comprised flowering plant species richness, grazing, and fertilization as fixed factors. We did not include mowing to avoid multicollinearity, because mowing was significantly positively correlated with fertilization and significantly negatively correlated with grazing for our study grassland plots (see Table SM2). Plot (field site, which was nested in bioregion, i.e., Exploratory) was used as random factor and year (2017 & 2018) as fixed factors in all models. Data distribution (Gaussian) and residuals were checked for normality and homogeneity of variances using Shapiro tests and graphical assessments. We always started with the most complex model which included all explanatory variables and eliminated all non‐significant effects using backward elimination (random and fixed; step function, *lmerTest package*) (Zuur et al., 2009) (Table 1). For the final model, *p*‐values for the fixed effects were calculated from F‐test based on Sattethwaite’s approximation and *p*‐values for the random effects were based on likelihood ratio tests. To compare differences in the variance explained by different models (i.e., including different explanatory variables) on the same response variables, we calculated R2‐values with the delta method using the pseudo‐R‐squared function (*MuMIn package*).

**TABLE 1:** Results of two separate generalized mixed effect models (GLMMs, F‐ and *p*‐values) analyzing the effect of (i) land‐use intensity (LUI) and flowering plant species richness (*PSR*), and (ii) grazing (*G*), fertilization (*F*), and flowering plant species richness (*PSR*) on bee abundance (i.e., number of individuals across species), bee species richness and taxonomic Shannon plant diversity in larval pollen provisions of all trap‐nesting solitary bee species. We did not include mowing to avoid multicollinearity, because mowing was significantly positively correlated with fertilization and significantly negatively correlated with grazing for our study grassland plots (see Table SM2). Year (2017 and 2018) was included as additional fixed factor and plot nested in bioregion as random factor in all models. Note that bee species identity (*ID*) was additionally included in the model on plant taxonomic diversity in larval pollen provisions of all seven trap‐nesting solitary bee species, but that we excluded bee species with less than 5 samples (*O*. *leaiana* & *Hylaeus* spp.). *p*‐values for the fixed effects included in the most parsimonious model were calculated from F‐tests based on Sattethwaite’s approximation. To compare differences in variance explained by different final models we calculated *R²*‐values (fixed effects: marginal *R²*: *_m_R²*; fixed and random effects: conditional *R²*: *_c_R²*). Plus signs indicate additive effects between fixed factors

Response variable			*_m_R²*	*_c_R²*	*F*	*P*			*_m_R²*	*_c_R²*	*F*	*p*
Bee variables												
Bee species abundance	LUI		0.14	0.36	7.31	.06	*PSR*		0.33	0.68	5.34	<.05
							*+G*				7.21	<.05
Bee species richness	LUI		0.20	0.54	8.17	<.01	*PSR*		0.18	0.51	3.92	<.01
							*+G*				6.24	<.01
Bee Shannon diversity	LUI		0.16	0.77	11.78	<.001	*PSR*		0.23	0.62	3.41	.08
Bee pollen provisions												
Taxonomic Shannon plant diversity	*ID*		0.11	0.42	24.32	<.01	*NA*		NA	NA	NA	NA

#### 2.4.1 Effects of land use on solitary bee species communities

We investigated how parameters related to land use and biogeographical region (Biodiversity Exploratories) affected trap nesting bees, that is, overall bee species diversity, richness, and abundance. Data of overall bee abundance were log‐transformed and bee species richness arcsine square‐root transformed to achieve statistical requirements.

To analyze how bioregion and/or LUI affected the community structures of bee species, permutation tests (PERMANOVA: Adonis, vegan package, permutations =10.000) were performed based on Bray–Curtis dissimilarities. Differences in community composition were visualized with nonmetric multidimensional scaling (NMDS). Environmental fitting (envfit, *vegan package*) was used to visualize effects of LUI on bee species community composition.

#### 2.4.2 Effects of land use on pollen foraging and bee–pollen plant networks

Plant diversity and composition in pollen provisions collected by different bee species was calculated as relative abundances of ASVs (amplicon sequence variant) taxonomic assignments. Bee species‐specific differences in the taxonomic composition of pollen provisions were investigated using PERMANOVAs based on Bray–Curtis dissimilarities and NMDS and environmental fitting for visualization.

Pollen‐based bee–plant interaction networks were displayed as bipartite networks. Plants visited for pollen collection by each bee species were included using relative abundances of ASVs after abundance filtering (min relative abundance 0.1%). We composed different interaction networks: (i) including all bee and plant species across bioregions and (ii) separate for each region. Networks for each bioregion were composed to display network patterns without risking forbidden links (Supplemental material Figure SM5). For all plant–bee networks, we calculated overall network specialization (H2’, network level) and specialization of individual bee species within networks (d’, standardized Kullback Leibler distance, species‐level) following (Blüthgen et al., 2006). However, we excluded bee species with less than 5 samples (*O*. *leaiana* & *Hylaeus* spp.) from this analysis to render our results more robust (Rivera‐Hutinel et al., 2012). H2’and d’ range from 0 to 1, with 0 representing no specialization and 1 high network specialization (H2’) or high species specialization (d’) (Blüthgen et al., 2006). We composed Patefield’s null models to assess levels of specialization (H2’) of our observed networks in relation to random networks.

#### 2.4.3 Effects of land use on pollen foraging, nutrition, and reproductive fitness in *Osmia bicornis*

We investigated how parameters related to land use and biogeographical region affected the taxonomic Shannon diversity and nutritional composition of larval pollen provisions as well as the number of larval brood cells in *Osmia bicornis*. Nutritional composition was measured as the total fatty acid (FA) concentration, the total amino acid (AA) concentration, as well as the total essential AA concentration and the AA‐to‐FA ratio (which equals the protein to lipid P:L ratio) in larval pollen provisions. To investigate how parameters related to land use affected the Shannon pollen diversity in larval provisions of *O*. *bicornis* (*n *= 90), we again composed two separate GLMMs with LUI, flowering plant species richness and year used as fixed factors in one model and flowering plant species richness, grazing, fertilization and year as fixed factors in the other model. Again plot (field site nested in Bioregion, i.e., Exploratory) was used as random factor in all models. To examine effects of bioregions and LUI on the plant taxonomic composition of *O*. *bicornis* larval provisions, we conducted PERMANOVAS, NMDS and environmental fitting. We additionally composed pollen‐based *O*. *bicornis*‐plant interaction networks based on ASVs found in *O*. *bicornis* larval provisions in relation to the three different LUI categories (see above).

Effects of all land‐use variables on pollen provision nutrients, that is, total FA, total AA, and essential AA content as well as the AA:FA ratio were analyzed using separate GLMMs as described above. To investigate how plant community composition affected the nutritional composition of FA and AA (in μg/mg pollen) of pollen collected by *O*. *bicornis*, we analyzed correlation patterns between plant taxa (agglomerated up to genus level) and single FAs or single AAs using the corrplot package and two matrices, one based on plant taxa and one based on FA or AA concentrations, respectively. Correlation coefficients between plant taxa and nutrients were calculated to assess correlation strength and direction (positive or negative). Significant correlations were adjusted for multiple testing by Benjamini–Hochberg corrections. To finally assess the effect of all land‐use variables on single FA, single AA, and single essential AA concentrations, separate GLMMs followed by correlation tests were performed. LUI, flowering plant species richness, and year were used as fixed factors in the first set of models and flowering plant species richness, grazing, fertilization, and year were used as fixed factors in the second set of models. Plot (field site) nested in bioregion (Exploratory) was used as random factor in all models.

Finally, land‐use‐induced differences in reproductive fitness (i.e., number of *O*. *bicornis* larval brood cells) were analyzed using separate GLMMs as described above followed by correlation tests.

## 3 RESULTS

### 3.1 Effects of land use on solitary bee species communities

**FIGURE 1:**
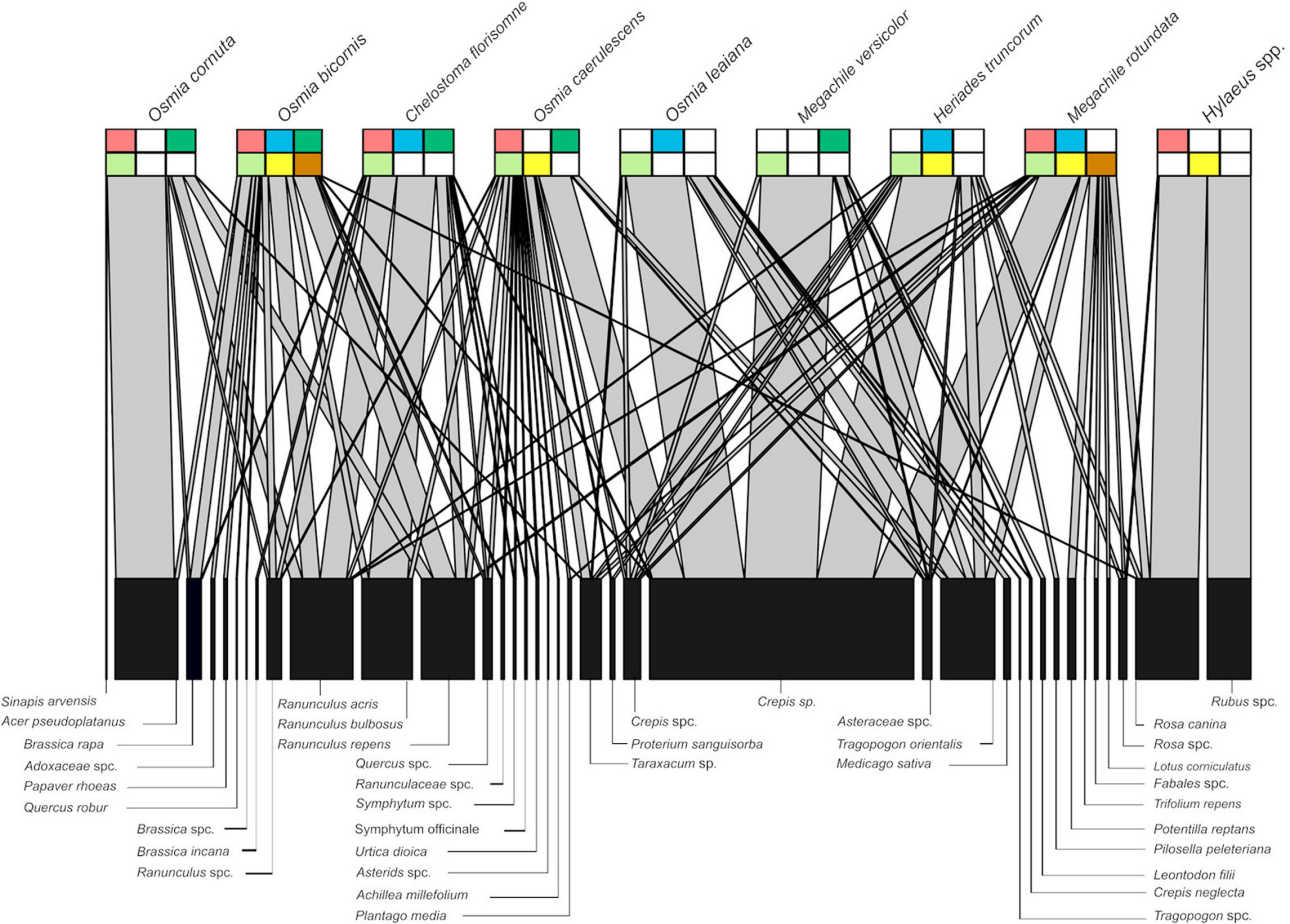
Bipartite network showing interactions between trap nesting solitary bee species and plant species based on larval pollen provisions sampled from nests installed at plots differing in land‐use intensity (LUI: represented by categories: low, intermediate, and high) in three biogeographical regions in Germany (Exploratories: Swabian Alb, Hainich‐Dün and Schorfheide‐Chorin) (assignment of ASVs up to species level). Plant species were included if they occurred in relative abundances of ≥1% in the respective dataset. *Osmia cornuta* is represented with 7 nest chambers, *O*. *bicornis* with 90, *Chelostoma florisomne* with 7, *Megachile rotundata* with 11, *Heriades truncorum* with 7, *O*. *caerulescens* with 15 and *O*. *leaiana* with 3, *M*. *versicolor* with 7 and *Hylaeus* spp. with 3. Colored bars below bee species show occurrence of each bee species at plots differing in land‐use intensity and in geographical regions; top bars indicate geographical distributions: red: Schorfheide‐Chorin, blue: Swabian Alb, darkgreen: Hainich‐Dün; bottom bars indicate land‐use intensity (LUI): lightgreen: low, yellow: intermediate, brown: high; white bars indicate that a species was absent from a specific bioregion or land‐use intensity category

In total, we found 285 occupied reed sticks and 1544 well‐developed bee individuals, that is, larvae which were not affected by fungi, parasitic insects, or nematodes, across all three bioregions. Overall bee species abundance, bee species richness, and Shannon diversity of bee species differed between bioregions (Table SM3). Some bee species were only found in specific regions, but not in others (Figure 1). Specifically, *Heriades truncorum* and *Osmia leaiana* were only found in the Swabian Alb. *Megachile versicolor* was exclusively found in the Hainich‐Dün, while *Hylaeus* were exclusively found in the Schorfheide. *O. cornuta* and *O. caerulescens* were both found in the Schorfheide and Hainich, while *M. rotundata* was found in the Schorfheide and the Swabian Alb. *Chelostoma florisomne* and *O. bicornis* were the only two species found across all bioregions (Figure 1).

Variation in bee species abundance, richness, and diversity were best explained by land‐use intensity (LUI) (Table 1). Bee species richness and Shannon diversity both significantly decreased with increasing land‐use intensity, while bee species abundance tended to decrease with increasing LUI (Table 1, Figure SM2). Both richness and abundance of bees significantly increased with increasing flowering plant species richness and with grazing intensity (Table 1). The Shannon diversity of bees also tended to increase with increasing flowering plant species richness (Table 1).

Bee community composition differed between bioregions (PERMANOVA: *R²* = 0.17, *p* < .05) and with LUI (*R²* = 0.11, *p* = .07) (Figure SM3). *Megachile rotundata* and *O*. *bicornis* were the only bee species found across the entire land‐use intensity gradient (Figure 1).

### 3.2 Effects of land use on pollen foraging and bee‐pollen plant networks

Sequencing of pollen samples generated on average 18,672 quality‐filtered ITS2 reads (range from 6429 to 98.819), in total 2.083.229 reads for the whole study. We found 267 taxonomic assignments on plant species level and 177 on genus level (Table SM4). Pollen taxonomic composition varied between bee species (PERMANOVA: *R²* = 0.021, *p* < .001, envfit: *R²* = 0.44, *p *< .001, Figure SM4) and with changes in flowering plant species richness (PERMANOVA: *R²* = 0.012, *p* < .57, envfit: *R²* = 0.08, *p *= .06).

The pollen‐based interaction network including all bee and plant species was generalized across all three bioregions (*H_2_’* = 0.407, Figure 1) and significantly different from random interactions (Table SM7). Interaction networks were similarly generalized within each bioregion (Swabian Alb: *H_2_’* = 0.499, Schorfheide‐Chorin: *H_2_’* = 0.480, Hainich‐Dün: *H_2_’* = 0.535, Figure SM5) and also significantly different from random interactions (Table SM7). Five bee species (*C*. *florisomne* (*n* = 7), *H*. *truncorum* (*n* = 7), *Hylaeus* spp. (*n* = 3), *M*. *versicolor* (*n* = 7), and *O*. *leaiana* (*n* = 3)) collected pollen from mostly one plant family (Figure 1), that is, Asteraceae in *M*. *versicolor* (*94*.*71%* of pollen taxa in larval provisions; *d’* = 0.60), *O*. *leaiana* (*90*.*14%*), and *H*. *truncorum* (*83*.*69%*; *d’* = 0.67); Ranunculaceae in *C*. *florisomne* (79.22%, *d’* = *0*.*49*) and Rosaceae in *Hylaeus* spp. (*94*.*63%*). Provisions of the other four bee species (*O*. *bicornis* (*n* = 90), *O*. *caerulescens* (*n* = 15), *O*. *cornuta* (*n* = 7), and *M*. *rotundata* (*n* = 11)) comprised pollen from between 6 and 10 different abundant plant families (Figure 1, Table SM4). Besides herbal species, several bee species also collected pollen from tree species, for example, *Acer pseudoplatanus (O*. *cornuta*, *O*. *bicornis)* and *Quercus robur* (*O*. *cornuta*, *O*. *bicornis*, *M*. *rotundata*, *C*. *florisomne*, and *O*. *caerulescens*). Both tree species were found in proximity to field sites (B. Peters, personal observation).

### 3.3 Effects of land use on pollen foraging, nutrition, and reproductive fitness in *Osmia bicornis*

**FIGURE 2:**
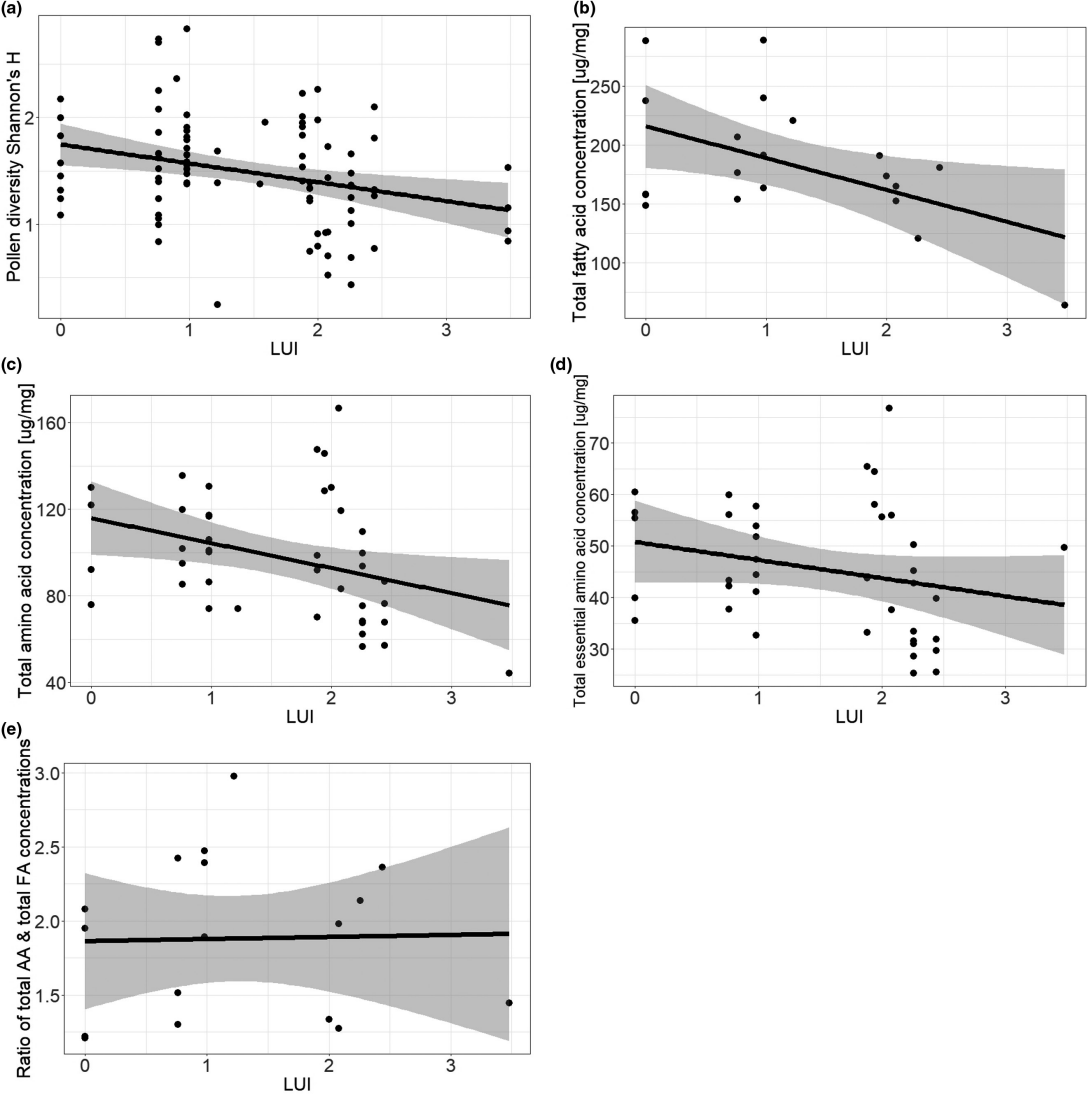
Effect of land‐use intensity (LUI) on (a) plant taxonomic Shannon diversity, (b) total fatty acid (*FA*) concentration (c) total amino (*AA*) acid concentration, and (d) total essential AA concentration and (e) the ratio of total *FA* to total *AA* in *Osmia bicornis* larval pollen provisions sampled from nests installed at plots differing in land‐use intensity (LUI) in three biogeographical regions in Germany (Exploratories: Swabian Alb, Hainich‐Dün and Schorfheide‐Chorin). Plant diversity is based on revealed ASVs (Amplicon sequent variants) per bee nest

The Shannon plant diversity of *O*. *bicornis* larval pollen provisions significantly decreased with increasing LUI (Table 2; Figure 2) and increased with flowering plant species richness (Table 2). Moreover, the relative abundance of Ranunculaceae and Brassicaceae plant species as well as of Sapindaceae, Rosaceae, and Papaveraceae (Table SM5 and Figure SM7) increased with increasing LUI. Land‐use intensity furthermore significantly affected pollen taxonomic composition in *O*. *bicornis* provisions (PERMANOVA: *R* ^2^ = 0.02, *p* < .01, Figure SM7 and SM8), while bioregion tended to affect the plant taxonomic composition of *O*. *bicornis* larval pollen provisions (PERMANOVA: *R* ^2^ = 0.09, *p* = .08).

**TABLE 2:** Results of two separate generalized mixed effect models (GLMMs, F‐ and *p*‐values) analyzing the effect of (i) land‐use intensity (LUI) and flowering plant species richness (*PSR*), and (ii) grazing (*G*), fertilization (*F*), and plant species richness (*PSR*) on taxonomic Shannon plant diversity of *O*. *bicornis* larval pollen provisions, the abundance of *O*. *bicornis* larval brood cells, as well as total fatty acids, total amino acids and the sum of total essential amino acids in *O*. *bicornis* larval pollen provisions. We did not include mowing to avoid multicollinearity, because mowing was significantly positively correlated with fertilization and significantly negatively correlated with grazing for our study grassland plots (see Table SM2). Year (2017 and 2018) was included as additional fixed factor and plot nested in bioregion as random factor in all models. *p*‐values for the fixed effects included in the most parsimonious model were calculated from F‐tests based on Sattethwaite’s approximation. To compare differences in variance explained by different final models we calculated *R²*‐values (fixed effects: marginal *R²*: *mR²*; fixed and random effects: conditional *R²*: *cR²*). Plus signs indicate additive effects between fixed factors

Response variable		*_m_R²*	*_c_R²*	*F*	*p*		*_m_R²*	*_c_R²*	*F*	*p*
*Osmia bicornis*—foraging, fitness, and pollen nutrient‐related variables										
Taxonomic Shannon plant diversity	LUI	0.07	0.12	6.22	<.05	*PSR*	0.11	0.39	8.07	<.001
Larval brood cells	LUI	0.14	0.31	5.89	.07	*PSR*	0.09	0.29	8.01	<.01
Total fatty acids	LUI	0.09	0.31	7.12	<.05	*G*	0.12	0.31	6.12	<.01
Total amino acids	LUI	0.12	0.31	10.09	.06	*PSR*	0.24	0.42	3.01	.09
						*+G*			4.98	.07
Total essential amino acid	LUI	0.20	0.28	8.07	<.05	*PSR*	0.12	0.48	3.61	.07
						*+G*			5.22	.057

Pollen‐based interaction networks between *O*. *bicornis* and plant species visited differed between low, intermediate, and high LUI (Figure SM6). The ten most abundant plant families in *O*. *bicornis* larval provisions were Ranunculaceae (~54% relative abundances of all *O*. *bicornis* samples), Brassicaceae (~19%), Sapindaceae (~4%), Fagaceae (~3%), Papaveraceae (~2%), Rosaceae (~3%), Asteraceae (~3%), Boraginaceae (~1%), Fabaceae (~1%), and Adoxaceae (~1%) (Table SM4). Relative abundances of Ranunculaceae pollen increased with increasing LUI (*low*: 38.59%; *intermediate*: 74.17%; *high*: 69.74%), while relative abundances of Brassicaceae pollen decreased with increasing LUI (*low*: 34.6%, *intermediate*: 1.28%, *high*: 1.42%) (Table SM5 and SM6 and Figure SM6). Likewise, relative abundances of Papaveraceae pollen (*low*: 1.2%; *intermediate*: 0.92%; *high*: 6.04%), Rosaceae (*low*: 2.61%; *intermediate*: 0.38%; *high*: 10.09%) increased with increasing land‐use intensity (Table SM5 and SM6, Figure SM7).

When analyzing the nutritional composition of *O*. *bicornis* larval pollen provisions, we found that both total FA concentrations and total AA concentrations significantly decreased with increasing LUI (Figure 2, Table 2, Table SM2), while the AA:FA ratio remained relatively constant across the entire land‐use intensity gradient (Figure 2). Additionally, total essential AA concentrations significantly decreased with increasing LUI (Table 2). Furthermore, total AA and total essential AA were positively affected by plant species richness and grazing, while total FA decreased with grazing intensity (Table 2).

While not all identified FAs were found in every pollen sample, 16 FAs were found across larval pollen provisions (Table SM9). Concentrations of individual FAs correlated with the abundance of specific plant species (Figure SM10). For example, relative abundances of some *Crepis* sp. in larval provisions were strongly positively correlated with linoleic acid (*LA)* and cerotic acid (*CA*) (Pearson correlation: *LA p* < .001, *CA p* < .001), while the relative abundance of *Ranunculus* sp. was positively correlated with α‐linolenic acid (*p* < .001) (Figure SM9).

Concentrations of six (out of the 16 analyzed) single FAs significantly decreased with increasing LUI (Table SM8 and SM9), while two FAs (myristic and margaric acid) increased with increasing LUI (Table SM8 and SM9). Concentrations of other FAs correlated positively or negatively with flowering plant species richness and/or fertilization (Table SM8 and SM9).

Concentrations of six essential amino acids, (threonine, valine, methionine, isoleucine, phenylalanine, and lysine) in *O*. *bicornis* larval pollen provisions significantly decreased with increasing LUI. Concentrations of other essential AAs were differentially affected by flowering plant species richness (valine and phenylalanine), grazing intensity (leucine, histidine), and/or both variables (arginine) (Table SM8 and SM9).

The number of larval brood cells, a proxy for reproductive fitness, significantly increased with increasing flowering plant species richness and tended to decrease with increasing LUI (Table 2).

## 4 DISCUSSION

Our study shows that land use can severely affect diversity, composition, and fitness parameters of trap‐nesting solitary bee species through altered interactions between bees and pollen resource plants and thereby their nutritional intake and reproductive success.

### 4.1 Effects of landuse on solitary bee communities and bee‐pollen plant networks

Bee species richness and Shannon diversity decreased with increasing land‐use intensity as did overall plant species richness in larval pollen provisions. In fact, we found bee species‐specific pollen compositions of larval provisions, indicating that diverse bee communities need diverse floral communities to meet all bee species‐specific dietary needs. Intensive land use can negatively impact on plant species richness as shown for the Biodiversity Exploratories (Weiner et al., 2014), where 34% of grassland plant species responded negatively to intensive land use (Busch et al., 2019), in particular to frequent mowing and fertilization (Socher et al., 2012). Reduced floral resource abundance and diversity, in turn, correlated with reduced bee species richness and diversity (Biesmeijer et al., 2006; Weiner et al., 2011, 2014). Our results indicate that the composition and diversity of trap nesting bee communities depended not only on overall resource diversity but also on the availability of specific pollen source plants. For example, *H*. *truncorum* and *M*. *versicolor*, which are all specialized on Asteraceae (Michener, 2007), mainly used pollen of *Crepis* sp. (>50%) for larval provisions and were almost exclusively present at sites where *Crepis biennis* was recorded, that is, on low‐intensity sites. The specialist *C*. *florisomne* was found across bioregions and mainly foraged on Ranunculaceae (79.22%) which were present at all study plots. Interestingly, *C*. *florisomne* was absent from field sites with intermediate or high land‐use intensity despite the occurrence of *Ranunculus* species, indicating that this bee species may require additional resources, for example, for nesting, or is additionally influenced by other factors, like, for example, pesticide exposure or competition (Centrella et al., 2020). By contrast, the two generalists *M*. *rotundata* and *O*. *bicornis* were found along the entire land‐use gradient.

Land‐use‐induced changes in plant diversity and composition can thus act as a filter on trap nesting bee communities. Flexible generalist bees, like *O*. *bicornis*, foraging on a wide spectrum of plants (Michener, 2007) may more easily find plant resources across landscapes differing in plant community composition and diversity (Mallinger et al., 2016). Such flexibility in foraging allows bee individuals to plastically respond to spatiotemporal changes in plant community composition and availabilities and to switch to alternative resources when necessary (Pornon et al., 2019). The less flexible specialist bees, for example, *H*. *truncorum* or *M*. *versicolor* (Michener, 2007), may in turn be restricted to specific landscapes and habitats providing suitable resources (Mallinger et al., 2016). This likely explains why intensively managed agricultural grasslands with severely reduced floral diversity harbor fewer bee species and impoverished bee communities (Grab et al., 2015; Mallinger et al., 2016), which does not only negatively affect bee populations (Renauld et al., 2016; Williams & Kremen, 2007), but also services provided as for example pollination (Goulson et al., 2015; Jauker et al., 2016; Potts et al., 2010).

Interestingly, we were able to observe effects of landuse on solitary bee communities at relatively small (plot/grassland site) scales, while previous studies did not find such effects at plot level (Fabian et al., 2014; Steckel et al., 2014). One limitation of our study is that we did not analyze our findings at larger scales. We can therefore not rule out that effects differ at different scales or that our results were additionally affected by variation in floral resource diversity and composition at the landscape scale. However, many (~85%) plant species revealed by metabarcoding were actually also found at plots or in the respective grasslands, indicating that female bees readily restrict pollen foraging to small scales if adequate pollen host plants are available (Ebeling et al., 2012).

### 4.2 Effects of land use on pollen foraging, nutrition, and reproductive fitness in *Osmia bicornis*

Interestingly, pollen composition of *O*. *bicornis* larval provisions changed with increasing land‐use intensity, revealing that *O*. *bicornis* shifted toward other plant species when the available plant community composition changed or appropriate floral resources were missing. For example, *O*. *bicornis* foraged on Ranunculaceae across bioregions and land‐use gradients, however, proportions of *Ranunculus bulbosus*, *R*. *acris* and *R*. *repens* varied between sites of high and low land‐use intensity: *R*. *bulbosus* decreased and *R*. *acris* and *R*. *repens* increased with increasing land‐use intensity. Also *Papaver rhoeas* and *Rosa canina* were more abundant in nests at intensively used field sites. These plant species were only observed in the environment surrounding plots (B. Peters, personal observation), which highlights the role of field borders and general landscape compositional heterogeneity for bees in intensively used agricultural landscapes (Hass et al., 2018).

The flexibility in resource use shown by *O*. *bicornis* enabled this species to collect sufficient resources and thus occur over the entire land‐use intensity gradient. Moreover, the ratio of amino acids (AA) to fatty acids (FA) (*AA*:*FA*) in *O*. *bicornis* larval pollen provisions remained relatively constant over the entire land‐use intensity gradient. The contents and ratios of these two macro‐nutrients are known to directly affect brood rearing success and immune defense in bees (Genissel et al., 2002; Human et al., 2007; Pirk et al., 2010; Ruedenauer et al., 2020; Tasei & Aupinel, 2008; Vaudo et al., 2015, 2016). It is therefore likely that *O*. *bicornis* regulates their intake and shows clear preferences for specific P:L ratios, as indicated by the relatively constant *AA*: *FA* (a proxy for P:L) ratio of 2:1 in larval provisions. In fact, *O*. *bicornis* appears to be able to maintain a specific nutritional intake (Vaudo, Stabler, et al., 2016) by shifting to alternate plant species. Interestingly, despite its flexibility, *O*. *bicornis* pollen provisions nevertheless contained overall lower concentrations of *FA*, *AA*, and essential *AA* at intensively managed sites. Pollen diversity in larval pollen provisions also decreased with increasing land‐use intensity as did the number of brood cells per nest. The change in nutritional content is most likely related to the observed changes in the spectrum of plants used as pollen source, suggesting that shifts in the taxonomic composition of *O*. *bicornis* larval pollen provisions, as found at high land‐use field sites, may nevertheless influence the suitability of pollen as larval food resource. Notably, we only investigated the composition of FA and AA, while overall pollen suitability and thus quality is determined by additional chemical compounds, such micro‐nutrients, sterols or plant secondary metabolites (PSMs) (Nicolson, 2011). In particular, PSMs strongly vary qualitatively and quantitatively among plant species and can both positively and negatively affect bee performance depending on their type and concentration (Palmer‐Young et al., 2019; Stevenson, 2020). There is some evidence that bees may be more likely to mitigate negative effects of PSMs through dilution when they have access to a broader spectrum of suitable pollen host plants (Eckhardt et al., 2014), which may further explain why some bee species were absent at sites with lower floral diversity. Besides pollen quality, pollen quantity can also increase with increasing floral diversity in the surrounding landscape, thus providing overall more resources to feed bees (as shown for the stingless bee species *Tetragonula carbonaria*: (Kaluza et al., 2018)).

Moreover, reduced overall floral abundance as frequently observed at sites with high land‐use intensities (Newbold et al., 2015) may force adult female bees to increase foraging trip duration to obtain sufficient resources (Danner et al., 2017; Westphal et al., 2006). This can in turn increase the vulnerability of nests to parasitism and predation (Goodell, 2003). Together, these factors may explain the observed decrease in the number of larval brood cells per nest. In fact, reduced floral diversity can reduce both the quantity and quality of allocated food (Kaluza et al., 2018; Trinkl et al., 2020). Changes in the nutritional quality of pollen diets may subsequently impair larval development, because plant pollen differ in their nutritional suitability even for generalist bees (Haider et al., 2013; Sedivy et al., 2011). Besides decreasing plant diversity and nutritional quality as well as shifting pollen composition, agri‐environmental factors may also indirectly impact on bee reproduction by increasing the risk of pesticide exposure (Centrella et al., 2020). Consequently, even highly flexible generalists, such as *O*. *bicornis*, may only be able to partly compensate for the loss of floral diversity.

## 5 CONCLUSION

Our findings highlight the importance of investigating land‐use‐induced changes in plant community composition in direct relation to foraging diversity, resource intake, and food nutritional quality, especially if we want to understand species‐specific responses and thus sensitivity to global change. Intensified land use can severely alter plant community composition and diversity. Our results show that such changes can also act as filter that select for species which are able to flexibly respond to changes in the resource landscape by shifting their resource use to alternative plants species, which enable them to maintain, for example, specific nutrient targets and thus (partly) compensate for the loss of floral diversity. Such species are likely generalists, but not every generalist might also be flexible enough. In fact, other hitherto neglected traits, such as cognitive flexibility, physiological tolerance, or broad nutritional niches, may best explain which species thrive and which perish in landscapes subject to global change.

## AUTHOR CONTRIBUTION

**Birte Peters:** Data curation (lead); Formal analysis (lead); Project administration (equal); Writing – original draft (equal); Writing – review & editing (equal). **Alexander Keller:** Conceptualization (equal); Formal analysis (supporting); Funding acquisition (equal); Project administration (equal); Resources (equal); Software (equal); Supervision (equal); Writing – review & editing (equal). **Sara Diana Leonhardt:** Conceptualization (equal); Formal analysis (supporting); Funding acquisition (equal); Project administration (equal); Resources (equal); Supervision (equal); Writing – review & editing (equal).

## CONFLICT OF INTEREST

The authors declare no conflict of interest.

## Supporting information

Supplementary Material

## Data Availability

The following data sets are available at BEXIS (https://www.bexis.uni‐jena.de) and are available online: Dataset ID/ Title 27228/Amino acids in pollen of Osmia bicornis larval provisions, DOI: https://doi.org/10.25829/bexis.27228‐4. 27227/Fatty acids in pollen of Osmia bicornis larval provisions, DOI: https://doi.org/10.25829/bexis.27227‐2. 27229/Plant species diversity based on ITS2 gene sequences of trap nesting solitary bee species, DOI: https://doi.org/10.25829/bexis.27229‐0. 27226/Trap nesting solitary bee species measured on all grassland VIPs, 2017–2019, DOI: https://doi.org/10.25829/bexis.27226‐4. 23586 & 24247 Vegetation Records for 150 Grassland EPs (2008–2018).
