# Supplementary material for: Diets maintained in a changing world: Does land‐use intensification alter wild bee communities by selecting for flexible generalists?
Source: Ecol Evol. 2022 May 15;12(5):e8919. doi: 10.1002/ece3.8919 (PMC9108308; doi:10.1002/ece3.8919)
Supplement: Supplementary file 1 — Supplementary Material [file ECE3-12-e8919-s001.docx]

**Supplemental material**

**Analytical details**

**Metabarcoding:**

Per reed, a maximum of 3-5 pollen provision samples were collected with sterile forceps, pooled per reed and weighed to measure the total amount of wet pollen provision per reed. Pooled samples were then transferred to autoclaved plastic tubes and directly frozen at -20°C. After removal, reeds were gently re-closed with remaining pollen to allow further larval development for a more detailed identification of adult bees after pupation.

The primer sequences including Illumina adapters were:

forward- AATGATACGGCGACCACCGAGATCTACAC-[8bp-i5-index]-CCTGGTGCTGGTATGCGATACTTGGTGTGAAT and reverse- CAAGCAGAAGACGGCATACGAGAT-[8bp-i7-index]-AGTCAGTCAGCCTCCTCCGCTTATTGATATGC-3.

After DNA extractions, performed with the Macherey-Nagel Nucleospin (Düren, Germany) kits for food we conducted PCR reactions in triplicates using 1-3 µl of template DNA in each reaction depending on DNA concentrations. The ThermoFisher Scientific PCR Phusion Master Mix was used with two indexed primers in a unique combination for each sample. Additionally, an appropriate quantity of PCR grade dH_2_O were used for every reaction. Samples were initially denatured at 95◦C for four minutes, then amplified with 37 cycles of 95◦C for 40 seconds, 49◦C for 40 seconds and 72◦C for 40 seconds. The program ended with a step of 72◦C for 5 minutes for final extension. After PCR, triplicates were pooled for each sample individually. A target of ~470–480 bp was amplified by the primers, which was then controlled for each sample by gel electrophoresis of PCR products in a 1.5% agarose gel. Using the Invitrogen SequalPrep Plate Normalization Kit (ThermoFisher Scientific, Life Technologies, Carlsbad, CA, USA) PCR DNA amounts were normalized between samples. We used the BioAnalyzer 2200 (Agilent, Santa Clara, USA) with High Sensitivity DNA Chips for library quality checks for pools of 96 samples. Pools were cleaned up using AMPure beads (Agilent Technologies, Santa Clara, CA, USA) to remove DNA fragments which are smaller than our amplicon. To quantify concentrations, we used a Qubit II Flurometer with the dsDNA High-Sensitivity Assay Kit (ThermoFisher Scientific, Life Technologies, Carlsbad, CA, USA). A final library pool was created following the Illumina MiSeq guidelines and was supplemented with 5% PhiXv3 (Illumina, 2016) and loaded into 500 cycle reagent Illumina Miseq cartridges along with the respective read 1 and read 2 sequencing primers.

All samples were sequenced in-house on a Miseq platform in the Department of Human Genetics of the University of Würzburg, Germany. We used USEARCH v11.0.667 (Edgar, 2013, 2016) to merge forward with corresponding reverse reads, accepted if longer than 200bp and passing quality filtering (Emax=1, no ambiguous base pairs) (Edgar & Flyvbjerg, 2015). For determination of amplicon sequence variants (ASVs), we deduplicated, sorted by length, denoised and chimera-filtered reads over all samples and removed singletons with the same software. ASVs were taxonomically classified using first global alignment searches using USEARCH (>97% identity) against a reference database containing all plant species known for the Biodiversity Exploratories created with the BCdatabaser software (Keller et al., 2020). Then for unclassified ASVs, we performed first a search against a similarly created reference database of all German plants (Ankenbrand, Keller, Wolf, Schultz, & Förster, 2015; Keller et al., 2014), then if still not classified followed by a hierarchical classification with SINTAX. Reads of each sample were mapped back to ASVs to receive counts of taxa per sample. Samples were removed from analysis in case they had less than 1500 reads after quality filtering (Sickel et al., 2015).

**Amino acid analysis:**

For the amino acid analysis, approximately 5.74 (± 4.38) mg pollen per sample were mixed with 200 µl of 6N HCl, boiled at 100°C for 4 h and then cooled down to room temperature and centrifuged for 10 min. The supernatant was transferred to a fresh plastic tube and boiled at 100°C to evaporate water. All samples were re-dissolved in fresh water three times and centrifuged again. Then, 100 μl of the supernatant were mixed with 20 µl of 12.5 % sulphosalicylic acid and extracted at ~5°C for 30 min, followed by brief mixing and centrifuging for 10 min. Finally, 100 μL of the supernatant were mixed with 100 μL sample rarefaction buffer in a fresh microcentrifuge tube, filtered and centrifuged for 5 min. The filtrate was analyzed by IEC. The IEC was equipped with a high-pressure PEEK column and an autosampler. Lithiumcitrate buffers (Laborservice Onken) with different pH values were used as eluents. After elution amino acids were stained with ninhydrine (Laborservice Onken) and measured with a photometer. We used an external standard (physiological calibration standard, Laborservice Onken GmbH, Gründau, Germany) for amino acid quantification, which contained all proteinogenic amino acids besides glutamine und asparagine, which were added manually prior to running standards and samples. Note that tryptophan is destroyed in HCl and could therefore not be analyzed with our approach.

**Fatty acid analysis:**

For the fatty acid analysis, samples of 12.18 (± 5.52) mg pollen were combined with 1 ml chloroform/methanol (2:1 V/V, both Sigma-Aldrich, Taufkirchen, Germany) and non-soluble fractions were discarded after 24 h incubation (300 rpm shaking) at 60°C. Separation of bound and free fatty acids was performed with 3 ml SiOH polypropylene-columns (CHROMABOND, 500mg, Trott, Germany) after adding 20 µl of nonadecanoic acid in methanol (0.2 mg/ml, both Sigma-Aldrich) as internal standard. Columns were pre-conditioned with 2 column equivalents (CE) of hexane (Merck). One ml pollen extract was then loaded onto the column. To eluate di-and trigylcerides, 4 ml isooctane/ethyl acetate (10:1, Sigma-Aldrich) and 5 ml isooctane/ethyl-acetate (3:1) were used, respectively. Free fatty acids were extracted with 6 ml isooctane/ethyl-acetate/acetic acid (75:25:2). All fractions were pooled, dried under CO2 and resolved in 250 μl dichloromethane/methanol (2:1). The pooled extract was completely evaporated under CO_2_ before adding 20 μl derivatizing agent (TMSH, Arcos Organics, New Jersey, USA).

We analyzed samples using a gas chromatograph coupled to a mass selective spectrometer (GCMS: 5975C intert XL MSD, Agilent Technologies, Santa Clara, USA). The GC was equipped with a DB-5 fused silica capillary column (30 m × 0.25 mm ID; df = 0.25 μm; Agilent Technologies, USA). The temperature program used started at initially 60°C, hold for one minute. Temperature was then increased by 15°C/min to 150°C and held for 10 min, before heating up to 320°C at 10°C/min, which was held for 10 min. We used helium as carrier gas with a constant flow of 2.89 ml/min. Injection temperature was 300°C, held for 1 min (splitless mode). Electron impact mass spectra (EI-MS) were recorded at 70 eV and 250°C. ChemStation (software package Agilent Technologies, Boblingen, Germany) was used for data acquisition. We analyzed fatty acids based on their mass spectra, retention times and through comparison with synthetic standards (Sigma-Aldrich, Munich, Germany). The internal standard nonadecanoic acid was used for quantification.

**Tables**

**Table SM1:** Plots (**Plot_ID**) of the three Biodiversity Exploratories (Schwabian Alb (AEG), Hainich-Dün (HEG) and Schorfheide Chorin (SEG)) included in our study; land-use intensity index (*LUI*) values based on grazing (**G**), mowing (**M**) and fertilization (**F**) are provided for each plot, as are the number of individuals per bee species collected from nests of different trap nesting bees (*Chelostoma florisomne (Cf)*, *Heriades truncorum (Ht)*, *Osmia bicornis (Ob)*, *Osmia caerulescens (Oca)*, *Osmia cornuta (OCo)*, *Megachile rotundata (Mr)*, *Megachile versicolor (Mv)*).

| **Plot_ID** | **G** | **M** | **F** | **LUI** | **Cf** | **Ht** | **Ob** | **OCa** | **OCo** | **Mr** | **Mv** |
| --- | --- | --- | --- | --- | --- | --- | --- | --- | --- | --- | --- |
| AEG1 | 0 | 1.91 | 2.32 | 2.06 |  | 11 |  | 6 |  | 9 |  |
| AEG2 | 0 | 2.87 | 9.23 | 3.48 |  |  | 41 |  |  | 2 |  |
| AEG3 | 0 | 1.91 | 0 | 1.38 | 3 |  |  |  |  |  |  |
| AEG4 | 1.24 | 0.96 | 1.58 | 1.94 |  |  | 53 |  |  |  |  |
| AEG5 | 1.01 | 0.96 | 1.59 | 1.88 | 23 |  | 17 | 9 |  | 6 |  |
| AEG6 | 0.76 | 0.96 | 0.8 | 1.59 |  | 9 | 6 | 7 |  | 15 | 6 |
| AEG7 | 0.34 | 0 | 0 | 0.59 | 27 |  |  |  |  |  |  |
| AEG8 | 0.4 | 0.96 | 0 | 1.16 |  | 3 | 3 |  |  |  |  |
| AEG9 | 0.82 | 0 | 0 | 0.9 |  | 6 | 7 |  |  | 11 |  |
| HEG1 | 0 | 1.91 | 10.16 | 3.47 |  |  | 3 |  |  | 3 |  |
| HEG2 | 0.2 | 1.91 | 3.21 | 2.31 |  |  | 5 |  |  |  |  |
| HEG3 | 0.2 | 1.91 | 3.21 | 2.31 |  |  | 3 |  |  |  |  |
| HEG4 | 0 | 0.96 | 3.13 | 2.02 | 4 |  | 3 |  |  |  |  |
| HEG5 | 0 | 1.91 | 2.43 | 2.08 |  | 8 | 58 | 18 |  |  | 2 |
| HEG6 | 0.17 | 0.96 | 4.84 | 2.44 |  |  | 66 |  |  |  | 1 |
| HEG7 | 5.13 | 0 | 0 | 2.26 |  |  | 36 | 5 | 12 |  |  |
| HEG8 | 1.48 | 0 | 0 | 1.22 | 17 |  | 23 | 42 | 11 | 10 | 25 |
| HEG9 | 0.58 | 0 | 0 | 0.76 | 5 | 4 | 247 | 24 | 4 |  | 15 |
| SEG1 | 0 | 0.96 | 0 | 0.98 |  |  | 36 |  |  | 17 | 2 |
| SEG2 | 1.17 | 0 | 0 | 1.08 |  |  | 9 |  |  |  |  |
| SEG3 | 1.23 | 0.96 | 0 | 1.48 |  | 9 | 4 | 16 | 4 |  |  |
| SEG4 | 0 | 0 | 0 | 0 |  |  | 80 | 24 | 29 | 6 |  |
| SEG5 | 0 | 0.96 | 0 | 0.98 | 9 |  | 232 |  | 14 |  |  |
| SEG6 | 3.04 | 0.96 | 0 | 2 |  |  | 27 | 10 | 6 |  |  |
| SEG7 | 2.32 | 0 | 0 | 1.52 |  |  | 8 |  |  |  |  |
| SEG8 | 3.76 | 0.96 | 0 | 2.17 |  |  | 6 | 12 |  |  |  |
| SEG9 | 2.39 | 0 | 0 | 1.55 |  |  | 8 |  | 6 |  |  |

**Table SM2:** Rank correlation matrix (Pearson) of all explanatory (land-use intensity 2017 (***LUI***); grazing (***G***); mowing (***M***); fertilization (***F***) and flowering plant species richness (***PSR***)) and response variables (bee species abundance; bee species richness (***BSr***); bee species Shannon diversity (***BSd***); *O. bicornis* larval brood cell numbers (N); taxonomic Shannon plant diversity, total fatty acid concentration (**FA**), total amino acid concentration (**AA**) and total essential amino acids (**EAA**) of *O.bicornis* larval pollen provisions) used in our analyses. Asterisks indicate significant correlations: * *p <* 0.05, ** *p <* 0.01, *** *p* < 0.001, marginal significant correlations are marked with “**.**”

| **Explanatory variables** | **LUI** | **G** | **M** | **F** | **PSR** | **BSr** | **BSd** |  |
| --- | --- | --- | --- | --- | --- | --- | --- | --- |
| LUI |  |  |  |  |  |  |  |  |
| G | 0.24 |  |  |  |  |  |  |  |
| M | **0.58**** | **-0.38.** |  |  |  |  |  |  |
| F | **0.73***** | -0.34 | **0.53*** |  |  |  |  |  |
| PSR | **0.51**** | 0.06 | **-0.35.** | -0.27 |  |  |  |  |
| **Response variables (Bee communities)** | | | | | | | | |
| Bee species abundance | **-0.38.** | -0.20 | -0.19 | -0.19 | **0.42*** | **0.67***** | **0.60**** |  |
| Bee species richness | **-0.44*** | -0.11 | -0.29 | -0.28 | **0.46*** |  | **0.76***** |  |
| Bee Shannon diversity | **-0.39*** | -0.29 | -0.07 | -0.16 | **0.33.** |  |  |  |
| **Response variables (Bee provisions)** | | | | | | | | |
| Taxonomic Shannon plant diversity | -0.14 | -0.11 | -0.02 | -0.04 | -0.13 |  |  |  |
| **Response variables (*Osmia bicornis* – foraging, fitness & pollen nutrients)** | | | | | | | | |
| Taxonomic Shannon plant diversity | **-0.27**** | **-0.12** | **-0.21*** | **-0.21*** | **0.12.** |  |  |  |
| N larval brood cells | **-0.07.** | **0.36.** | -0.17 | -0.19 | **0.12.** |  |  |  |
| Total FA | **-0.15*** | -0.08 | 0.27 | 0.16 | 0.42 |  |  |  |
| Total AA | **-0.43**** | -0.25 | -0.08 | **-0.38*** | -0.26 |  |  |  |
| Total EAA | **-0.32*** | **-0.31*** | 0.13 | -0.14 | -0.11 |  |  |  |

**Table SM3:** Mean ( ± standard deviation, SD) abundance, species richness and Shannon diversity of trap nesting bees collected from nests installed at plots differing in land-use intensity (LUI: represented by categories: low, intermediate and high) in three bioregions in Germany (Exploratories: Swabian Alb, Hainich-Dün and Schorfheide-Chorin).

| **Exploratory** | **Bee abundance**  **(Mean ± SD)** | **Richness**  **(Mean ± SD)** | **Diversity**  **(Mean ± SD)** |
| --- | --- | --- | --- |
| Swabian Alb | 7.8 ± 3.4 | 2.3 ± 1.3 | 1.2 ± 0.3 |
| Hainich-Dün | 17.3 ± 19.2 | 2.4 ± 1.1 | 0.9 ± 0.3 |
| Schorfheide-Chorin | 11.0 ± 17.4 | 1.9 ± 0.8 | 0.8 ± 0.3 |
| **LUI** |  |  |  |
| Low | 17.9 ± 21.1 | 2.6 ± 1.2 | 1.1 ± 0.3 |
| Intermediate | 7.6 ± 5.5 | 1.9 ± 0.9 | 0.9 ± 0.4 |
| High | 6.0 ± 7.1 | 1.5 ± 0.7 | 0.8 ± 0.4 |

**Table SM4:** Percentages of plant taxa in larval pollen provision samples collected from nests of different trap nesting bee species (*Chelostoma florisomne*, *Heriades truncorum*, *Hylaeus spp.*, *Osmia bicornis*, *Osmia caerulescens*, *Osmia cornuta*, *Osmia leaiana*, *Megachile rotundata*, *Megachile versicolor*) in three biogeographical regions in Germany along a land-use intensity gradient. Percentages are based on relative abundance of plant families. Values > 50% are marked in bold.

| **Order** | **Family** | ***Chelostoma forisomne*** | ***Heriades truncorum*** | ***Hylaeus* spp.** | ***Osmia bicornis*** | ***Osmia cearulescens*** | ***Osmia cornuta*** | ***Osmia leaiana*** | ***Megachile rotundata*** | ***Megachile versicolor*** |
| --- | --- | --- | --- | --- | --- | --- | --- | --- | --- | --- |
| Sapindales | Aceraceae | 0 | 0 | 0 | 0.03 | 0 | 0.11 | 0 | 0 | 0 |
| Dipsacales | Adoxaceae | 0.39 | 0.03 | 0.15 | 1.13 | 0.32 | <0.01 | 0.02 | 0.40 | 0.10 |
| Asparagales | Amaryllidaceae | 0 | 0 | <0.01 | 0.08 | 0.15 | 6.36 | 0 | 0 | 0 |
| Apiales | Apiaceae | 0.283 | 0.17 | 0.41 | 0.25 | 0.72 | 0.10 | 0.14 | 0.20 | 0.46 |
| Apiales | Apiales spc | 0 | <0.01 | 0 | <0.01 | 0 | 0 | 0 | <0.01 | 0.02 |
| Apiales | Araliaceae | 0.82 | 0.37 | 0.24 | 0.89 | 0.91 | 1.31 | 0.02 | 0.35 | 1.05 |
| Asterales | Asteraceae | 2.18 | **83.69** | 0.49 | 3.18 | **50.64** | 1.32 | **90.14** | **58.45** | **94.71** |
| Asterales | Asterales spc | <0.01 | 0.05 | 0 | <0.01 | 0.18 | 0 | 0.04 | 0.06 | 0.08 |
| Fagales | Betulaceae | 0 | 0 | 0 | 0 | 0 | 0 | 0 | 0 | 0 |
| Boraginales | Boraginaceae | 2.24 | 0.02 | 0 | 1.37 | 2.41 | 0.10 | <0.01 | 0.06 | <0.01 |
| Boraginales | Boraginales spc | 0.02 | 0 | 0 | <0.01 | <0.01 | 0 | 0 | <0.01 | 0 |
| Brassicales | Brassicaceae | 1.08 | 0.23 | 0.09 | 19.52 | 3.15 | 3.25 | 0.04 | 1.67 | 0.36 |
| Caryophyllales | Caryophyllaceae | 0.15 | <0.01 | 0.05 | 0.10 | 1.05 | 0 | 0.02 | 0.02 | <0.01 |
| Caryophyllales | Caryophyllales spc | <0.01 | 0 | 0 | <0.01 | <0.01 | <0.01 | 0 | 0 | <0.01 |
| Solanales | Convolvulaceae | <0.01 | 0 | 0 | 0 | <0.01 | 0 | 0 | 0 | <0.01 |
| Cupressales | Cupressaceae | 0 | 0 | 0 | <0.01 | 0 | 0 | 0 | 0 | 0 |
| Poales | Cyperaceae | 0.52 | 0 | 0 | 0.01 | 0 | 0 | 0 | <0.01 | 0 |
| Dipsacales | Dipsacales spc | 0.06 | <0.01 | 0 | 0.16 | 0.02 | 0 | 0 | 0.02 | <0.01 |
| Ericales | Ericaceae | 0.03 | 0.09 | 0.29 | 0.05 | 0.1 | <0.01 | 0.02 | 0.09 | 0.03 |
| Fabales | Fabaceae | 0.79 | 0.06 | 1.43 | 1.21 | 0.34 | 0.31 | 0.02 | 4.64 | 0.77 |
| Fabales | Fabales spc | 0.08 | 0 | 0.64 | 0.01 | <0.01 | 0.03 | <0.01 | 3.87 | 0.02 |
| Fagales | Fagaceae | 2.43 | 0 | <0.01 | 3.29 | 6.091 | 0.20 | 0 | <0.01 | 0 |
| Fagales | Fagales spc | 0.04 | 0 | 0 | 0.12 | 0.41 | 0 | 0 | <0.01 | 0 |
| Geraniales | Geraniaceae | 0.03 | 0 | 0 | <0.01 | <0.01 | 0 | 0 | <0.01 | 0 |
| Geraniales | Geraniales spc | 0 | 0 | 0 | <0.01 | 0 | 0 | 0 | <0.01 | 0 |
| Malpighiales | Hypericaceae | 0 | 0 | 0.05 | <0.01 | 0 | 0 | <0.01 | <0.01 | 0 |
| Fagales | Juglandaceae | 0.06 | 0 | 0 | 0.41 | 0.04 | 0.01 | <0.01 | 0.01 | 0.04 |
| Lamiales | Lamiaceae | 0.02 | 0 | 0 | 0 | 0.40 | 0 | 0.01 | 0 | 0 |
| Lamiales | Lamiales spc | 0.02 | 0 | 0 | 0.31 | 0.37 | <0.01 | 0 | <0.01 | 0 |
| Liliopsida | Liliopsida spc | <0.01 | 0 | 0 | 0 | 0 | 0 | 0 | 0 | <0.01 |
| Myrtales | Lythraceae | 0 | 0 | 0 | <0.01 | 0 | 0 | 0 | 0 | 0 |
| Malvales | Malvaceae | 0.03 | 0.08 | <0.01 | 0.01 | 0 | <0.01 | 0 | 0.34 | 0 |
| Lamiales | Oleaceae | <0.01 | 0 | 0.01 | 0.01 | 0 | 0 | 0 | 0 | 0 |
| Myrtales | Onagraceae | 0 | 0 | 0 | <0.01 | 0 | <0.01 | 0 | <0.01 | 0.03 |
| Lamiales | Orobanchaceae | <0.01 | 0 | 0.04 | 0 | <0.01 | 0 | 0 | 0.02 | 0.04 |
| Saxifragales | Paeoniaceae | 0.071 | 0 | 0 | 0.55 | 0.03 | 0.09 | 0 | 0.02 | 0.04 |
| Ranunculales | Papaveraceae | 5.712 | 0 | 0 | 1.89 | 4.82 | 0.11 | 0.03 | 0.05 | 0.04 |
| Pinales | Pinaceae | <0.01 | 0 | 0 | <0.01 | 0 | <0.01 | 0 | 0 | 0 |
| Lamiales | Plantaginaceae | 0.11 | 0.09 | 0.22 | 0.31 | 3.10 | 0.09 | 0.04 | 1.09 | 0.02 |
| Poales | Poaceae | 0.4 | 0.03 | 0.01 | 1.01 | 0.36 | <0.01 | 0.12 | 0.04 | 0.05 |
| Ericales | Primulaceae | 0 | 0 | 0 | 0 | 0 | 0 | 0 | 0.01 | 0 |
| Ranunculales | Ranunculaceae | **79.22** | 2.52 | 0.89 | **54.05** | 19.03 | 24.79 | 0.77 | 15.13 | 0.91 |
| Ranunculales | Ranunculales spc | 0.05 | <0.01 | 0 | 0.10 | 0.06 | 0 | 0 | 0.05 | 0 |
| Rosales | Rosaceae | 0.53 | 11.92 | **94.63** | 3.07 | 1.04 | 0.3 | 8.43 | 12.18 | 0.57 |
| Rosales | Rosales spc | 0.01 | 0.11 | 0.04 | <0.01 | <0.01 | <0.01 | <0.01 | 0.03 | <0.01 |
| Gentianales | Rubiaceae | 0 | <0.01 | 0 | 0 | <0.01 | 0 | 0 | 0.01 | 0 |
| Malpighiales | Salicaceae | 0 | 0 | 0 | <0.01 | 0 | 0 | 0 | 0 | 0 |
| Sapindales | Sapindaceae | 0.52 | 0 | 0.03 | 4.22 | 0.13 | **59.63** | 0.02 | 0.02 | 0.06 |
| Sapindales | Sapindales spc | 0 | 0 | 0 | <0.01 | 0 | 0 | <0.01 | 0 | 0 |
| Saxifragales | Saxifragales spc | 0.10 | 0 | 0 | 0.96 | 0.03 | 0.07 | 0 | 0.19 | 0.05 |
| sub:asterids | Asterids spc | 0.09 | 0.07 | 0.02 | 0.11 | 1.07 | 0.09 | 0.03 | 0.20 | 0.13 |
| sub:rosids | Rosids spc | 0.22 | 0.04 | 0.06 | 0.55 | 0.56 | 1.14 | 0 | 0.18 | 0.08 |
| Rosales | Urticaceae | 0.89 | <0.01 | 0.02 | 0.02 | 1.85 | 0.25 | <0.01 | 0.08 | <0.01 |
| Viridiplantae spc | Viridiplantae spc | 0.75 | 0.41 | 0.11 | 0.93 | 0.56 | 0.28 | 0.03 | 0.45 | 0.3 |
|  |  |  |  |  |  |  |  |  |  |  |

**Table SM5:** Percentages of plant taxa (family level) in larval pollen provision samples collected from nests of *Osmia bicornis* in three bioregions in Germany along a land-use intensity gradient (with low, intermediate (Inter) and high land-use intensity). Percentages are based on relative abundances [%] of the 10 most abundant plant families (marked in bold).

| **Order** | **Family** | **Abundance**  **Low[%]** | **Abundance**  **Inter[%]** | **Abundance**  **High[%]** |
| --- | --- | --- | --- | --- |
| Ranunculales | **Ranunculaceae** | **38.99** | **74.68** | **69.74** |
| Brassicales | **Brassicaceae** | **34.90** | **1.29** | **1.43** |
| Sapindales | **Sapindaceae** | **3.87** | **3.70** | **6.51** |
| Asterales | **Asteraceae** | **3.45** | **3.94** | **0.67** |
| Rosales | **Rosaceae** | **2.62** | **0.38** | **10.09** |
| Fagales | **Fagaceae** | **2.33** | **6.58** | **0.18** |
| Dipsacales | **Adoxaceae** | **1.84** | **0.27** | **0.25** |
| Saxifragales | Saxifragales spc | 1.75 | <0.05 | <0.05 |
| Boraginales | **Boraginaceae** | **1.70** | **1.31** | **0.29** |
| Ranunculales | **Papaveraceae** | **1.23** | **0.92** | **6.05** |
| Poales | Poaceae | 1.27 | 0.99 | 0.06 |
| Apiales | Araliaceae | 1.08 | 0.69 | 0.52 |
| Saxifragales | Paeoniaceae | 0.99 | <0.05 | <0.05 |
| Fagales | Juglandaceae | 0.74 | <0.05 | <0.05 |
| Fabales | **Fabaceae** | **0.55** | **1.87** | **2.82** |
| Lamiales | Plantaginaceae | 0.42 | 0.13 | 0.27 |
| rosids | rosids spc | 0.39 | 1.02 | 0.19 |
| Dipsacales | Dipsacales spc | 0.23 | <0.05 | 0.05 |
| Apiales | Apiaceae | 0.22 | <0.05 | <0.05 |
| Caryophyllales | Caryophyllaceae | 0.17 | <0.05 | <0.05 |
| asterids | asterids spc | 0.16 | 0.07 | <0.05 |
| Asparagales | Amaryllidaceae | 0.07 | 0.06 | <0.05 |
| Lamiales | Lamiales spc | <0.05 | 1.00 | <0.05 |
| Fagales | Fagales spc | <0.05 | 0.32 | <0.05 |
| Apiales | Apiaceae | <0.05 | 0.32 | 0.23 |
| Fagales | Betulaceae | <0.05 | 0.13 | <0.05 |
| Ranunculales | Ranunculales spc | 0.09 | 0.12 | 0.20 |
| Ericales | Ericaceae | 0.06 | <0.05 | <0.05 |

**Table SM6:** Percentages of plant taxa (species level) in larval pollen provision samples collected from nests of *Osmia bicornis* in three bioregions in Germany along a land-use intensity gradient (with low, intermediate (Inter) and high land-use intensity). Percentages are based on relative abundances [%] of the 10 most abundant plant species (marked in bold).

| **Order** | **Family** | **Genus** | **Species** | **Abundance**  **Low[%]** | **Abundance**  **Inter[%]** | **Abundance**  **High[%]** |
| --- | --- | --- | --- | --- | --- | --- |
| Sapindales | Sapindaceae | Acer | *Acer pseudoplatanus* | **3.86** | **6.51** | **0.37** |
| Dipsacales | Adoxaceae | Adoxaceae spc | Adoxaceae spc | 1.80 | 0.27 | 0.25 |
| Rosales | Rosaceae | Alchemilla | *Alchemilla vulgaris* | 0.11 | <0.05 | <0.05 |
| Asparagales | Amaryllidaceae | Allium | Allium spc | 0.07 | 0.06 | 0.18 |
| Poales | Poaceae | Alopecurus | *Alopecurus pratensis* | 1.13 | 0.09 | <0.05 |
| Apiales | Apiaceae | Anthriscus | *Anthriscus sylvestris* | 0.11 | 0.2 | 0.14 |
| Brassicales | Brassicaceae | Arabidopsis | *Arabidopsis thaliana* | 1.19 | 0.14 | <0.05 |
| asterids | asterids spc | asterids spc | asterids spc | 0.16 | 0.07 | <0.05 |
| Asterales | Asteraceae | Bellis | *Bellis margaritifolia* | <0.05 | 0.15 | 0.11 |
| Fagales | Betulaceae | Betula | *Betula pendula* | <0.05 | 0.13 | <0.05 |
| Asterales | Asteraceae | Bidens | *Bidens cronquistii* | 0.06 | <0.05 | <0.05 |
| Brassicales | Brassicaceae | Brassica | *Brassica rapa* | **19.63** | **0.555** | **0.82** |
| Brassicales | Brassicaceae | Brassica | Brassica spc | 7.06 | 0.1 | 0.157 |
| Brassicales | Brassicaceae | Brassicaceae spc | Brassicaceae spc | <0.05 | 0.05 | <0.05 |
| Brassicales | Brassicaceae | Hirschfeldia | *Hirschfeldia incana* | 6.49 | 0.21 | 0.33 |
| Ericales | Ericaceae | Calluna | Calluna spc | 0.05 | <0.05 | <0.05 |
| Ranunculales | Ranunculaceae | Caltha | Caltha spc | 0.08 | <0.05 | <0.05 |
| Apiales | Apiaceae | Chaerophyllum | *Chaerophyllum temulum* | 0.09 | <0.05 | 0.06 |
| Rosales | Rosaceae | Crataegus | *Crataegus monogyna* | <0.05 | 0.43 | <0.05 |
| Rosales | Rosaceae | Crataegus | Crataegus spc | 0.07 | <0.05 | <0.05 |
| Asterales | Asteraceae | Crepis | *Crepis neglecta* | <0.05 | 0.05 | <0.05 |
| Asterales | Asteraceae | Crepis | Crepis spc | <0.05 | 0.11 | <0.05 |
| Asterales | Asteraceae | Crepis | *Crepis vesicaria* | 0.94 | 2.89 | 0.34 |
| Poales | Poaceae | Dactylis | *Dactylis glomerata* | <0.05 | 0.70 | <0.05 |
| Dipsacales | Dipsacales spc | Dipsacales spc | Dipsacales spc | 0.26 | <0.05 | <0.05 |
| Dipsacales | Dipsacales spc | Dipsacales spc | Dipsacales spc | <0.05 | <0.05 | 0.05 |
| Fabales | Fabaceae | Fabaceae spc | Fabaceae spc | 0.06 | <0.05 | <0.05 |
| Fagales | Fagales spc | Fagales spc | Fagales spc | <0.05 | 0.32 | <0.05 |
| Apiales | Apiaceae | Heracleum | *Heracleum sphondylium* | <0.05 | 0.11 | <0.05 |
| Fagales | Juglandaceae | Juglans | Juglans spc | 0.74 | <0.05 | <0.05 |
| Lamiales | Lamiales spc | Lamiales spc | Lamiales spc | <0.05 | 1.00 | <0.05 |
| Asterales | Asteraceae | Leucanthemum | *Leucanthemum gallaecicum* | 0.12 | <0.05 | <0.05 |
| Boraginales | Boraginaceae | Myosotis | *Myosotis arvensis* | 1.12 | 0.58 | 0.26 |
| Saxifragales | Paeoniaceae | Paeoniaceae spc | *Paeoniaceae spc* | 0.99 | <0.05 | <0.05 |
| Ranunculales | Papaveraceae | Papaver | *Papaver rhoeas* | **1.07** | **0.71** | **4.42** |
| Ranunculales | Papaveraceae | Papaver | Papaver spc | 0.21 | 0.21 | 1.62 |
| Lamiales | Plantaginaceae | Plantago | *Plantago lanceolata* | <0.05 | 0.06 | <0.05 |
| Lamiales | Plantaginaceae | Plantago | *Plantago media* | 0.32 | <0.05 | <0.05 |
| Poales | Poaceae | Poaceae spc | Poaceae spc | 0.09 | 0.19 | <0.05 |
| Rosales | Rosaceae | Potentilla | *Potentilla reptans* | 0.79 | <0.05 | <0.05 |
| Rosales | Rosaceae | Poterium | *Poterium sanguisorba* | 0.36 | <0.05 | <0.05 |
| Fagales | Fagaceae | Quercus | *Quercus robur* | 0.28 | 3.05 | <0.05 |
| Fagales | Fagaceae | Quercus | Quercus spc | **2.04** | **3.52** | **0.16** |
| Ranunculales | Ranunculaceae | Ranunculaceae spc | Ranunculaceae spc | **1.63** | **1.79** | **0.82** |
| Ranunculales | Ranunculales spc | Ranunculales spc | Ranunculales spc | 0.07 | 0.12 | 0.20 |
| Ranunculales | Ranunculaceae | Ranunculus | *Ranunculus acris* | **14.30** | **18.81** | **26.1** |
| Ranunculales | Ranunculaceae | Ranunculus | *Ranunculus bulbosus* | **3.15** | **16.07** | **1.32** |
| Ranunculales | Ranunculaceae | Ranunculus | *Ranunculus repens* | **16.31** | **28.89** | **32.39** |
| Ranunculales | Ranunculaceae | Ranunculus | Ranunculus spc | **5.12** | **9.12** | **9.61** |
| Fabales | Fabaceae | Robinia | Robinia spc | <0.05 | 1.72 | <0.05 |
| Rosales | Rosaceae | Rosa | *Rosa canina* | **1.05** | **0.21** | **7.93** |
| Rosales | Rosaceae | Rosa | Rosa spc | 0.12 | 0.12 | 1.67 |
| rosids | rosids spc | rosids spc | Rosids spc | 0.39 | 1.02 | 0.19 |
| Saxifragales | Saxifragales spc | Saxifragales spc | Saxifragales spc | 1.75 | <0.05 | <0.05 |
| Caryophyllales | Caryophyllaceae | Silene | *Silene flos-cuculi* | 0.09 | <0.05 | <0.05 |
| Brassicales | Brassicaceae | Sinapis | *Sinapis arvensis* | 0.12 | 0.15 | 0.05 |
| Boraginales | Boraginaceae | Symphytum | *Symphytum officinale* | 0.24 | 0.26 | <0.05 |
| Boraginales | Boraginaceae | Symphytum | Symphytum spc | 0.26 | 0.47 | <0.05 |
| Asterales | Asteraceae | Taraxacum | Taraxacum spc | 1.37 | 0.5 | 0.07 |
| Asterales | Asteraceae | Tragopogon | *Tragopogon orientalis* | 0.29 | <0.05 | <0.05 |
| Asterales | Asteraceae | Tripleurospermum | *Tripleurospermum maritimum* | 0.41 | <0.05 | <0.05 |
| Lamiales | Plantaginaceae | Veronica | *Veronica chamaedrys* | <0.05 | 0.20 | <0.05 |
| Fabales | Fabaceae | Vicia | *Vicia faba* | 0.34 | 0.14 | <0.05 |
| Fabales | Fabaceae | Vicia | Vicia spc | <0.05 | 1.68 | 0.52 |
| Fabales | Fabaceae | Vicia | *Vicia villosa* | 0.10 | <0.05 | <0.05 |

**Table SM7:** Network specialization (*H_2_*‘) and p-values (*p*) of comparisons between observed networks and random networks (Patefield’s null model) and specialization of different trap nesting bee species (d’) across all bioregions and within each bioregion (Swabian Alb (ALB), Schorfheide- Chorin (SCH) and Hainich-Dün (HAI). Note that we excluded bee species with less than 5 samples (*O. leaiana* & *Hylaeus* spp.).

|  |  |  | ***Chelostoma florisomne*** | ***Heriades truncorum*** | ***Megachile rotundata*** | ***Megachile versicolor*** | ***Osmia bicornis*** | ***Osmia caerulescens*** | ***Osmia cornuta*** |
| --- | --- | --- | --- | --- | --- | --- | --- | --- | --- |
| ***All Bioregions*** | 0.407 | < 0.001 | 0.494 | 0.67 | 0.43 | 0.60 | 0.45 | 0.32 | 0.37 |
| ***ALB.*** | 0.499 | < 0.001 | 0.54 | 0.69 | 0.50 | NA | 0.57 | NA | NA |
| ***SCH*** | 0.480 | < 0.001 | 0.28 | NA | 0.69 | NA | 0.43 | 0.55 | 0.25 |
| ***HAI*** | 0.535 | < 0.001 | 0.49 | NA | NA | 0.71 | 0.51 | 0.47 | 0.61 |

**Table SM8:** Rank correlations (Pearson) of mean (in μg/mg larval provision) concentrations of single amino acids and fatty acids in larval pollen provisions of *Osmia bicornis* with different parameters related to land-use. Abbreviations as follows: ***LUI***: land-use intensity; ***G***: grazing; ***M***: mowing; ***F***: fertilization; ***PSR***: plant species richness. Asterisks indicate significant correlations: * *p <* 0.05, ** *p <* 0.01, *** *p* < 0.001. Essential amino acids are marked in bold-italic; fatty acids which were found across all pollen samples are marked in bold.

| **Response variables** | **Explanatory variables** | | |  |  |  |
| --- | --- | --- | --- | --- | --- | --- |
| **Single amino acids** | **LUI** | **G** | **M** | | **F** | **PSR** |
| Asparagine acid | -0.18 | -0.25 | 0.20 | | -0.06 | -0.12 |
| ***Threonine*** | **-0.37**** | **-0.32*** | 0.07 | | -0.13 | -0.13 |
| Serine | -0.40** | **-0.36*** | 0.11 | | -0.13 | 0.11 |
| Glutamic acid | **-0.30.** | **-0.31.** | 0.17 | | -0.15 | -0.16 |
| ***Proline*** | -0.01 | -0.07 | 0.18 | | -0.01 | -0.09 |
| Glycine | **-0.35*** | **-0.38*** | 0.13 | | -0.09 | -0.02 |
| Alanine | **-0.36*** | **-0.39*** | 0.15 | | 0.12 | -0.06 |
| ***Valine*** | **-0.46**** | **-0.32*** | 0.03 | | -0.28. | 0.13 |
| ***Methionine*** | **-0.54***** | **-0.28.** | -0.14 | | **-0.37*** | 0.08 |
| ***Isoleucine*** | **-0.48**** | **-0.40**** | 0.08 | | -0.24 | -0.08 |
| ***Leucine*** | **-0.28.** | **-0.40*** | 0.27 | | -0.01 | -0.19 |
| Tyrosine | **-0.40*** | **-0.45**** | 0.15 | | -0.04 | -0.15 |
| ***Phenylalanine*** | **-0.44**** | **-0.37*** | 0.06 | | -0.22 | 0.07 |
| Gamma amino butyric acid | -0.11 | -0.16 | 0.10 | | -0.04 | -0.18 |
| ***Lysine*** | **-0.47**** | **-0.36*** | 0.03 | | -0.21 | -0.11 |
| ***Histidine*** | -0.23 | -0.18 | 0.11 | | -0.21 | -0.04 |
| ***Arginine*** | -0.25 | **-0.28.** | 0.15 | | -0.09 | -0.09 |
| **Single fatty acids** | **LUI** | **G** | **M** | | **F** | **PSR** |
| **Caprylic (Octanoic) acid** | -0.18 | -0.21 | 0.03 | | -0.18 | -0.21 |
| **Glutaric (Pentanedioic) acid** | -0.47 | -0.19 | -0.32 | | -0.17 | -0.21 |
| **Pelargonic (Nonanoic) acid** | **-0.38*** | **-0.26** | -0.22 | | -0.24 | -0.16 |
| Adipic (Hexanedioic) acid | **-0.38*** | -0.21 | -0.11 | | -0.26 | **-0.50*** |
| **Capric (Decanoic) acid** | 0.18 | -0.30 | 0.23 | | **0.38*** | 0.22 |
| **Lauric (Dodecanoic) acid** | -0.04 | -0.15 | 0.11 | | -0.02 | -0.13 |
| **Azelaic (Nonanedioic) acid** | **-0.61**** | -0.29 | -0.37 | | -0.32 | 0.08 |
| Sebacic (Decanedioic) acid | -0.34 | -0.05 | -0.14 | | -0.31 | **-0.39*** |
| **Myristic (Tetradecanoic) acid** | 0.01 | -0.12 | -0.05 | | -0.05 | 0.23 |
| Methyltetradecanoic acid 1 & 2 | -0.14 | **0.43*** | **-0.55**** | | -0.34 | **0.51*** |
| Hexadecatrienoic acid | -0.17 | 0.06 | 0.19 | | 0.09 | 0.24 |
| Pentadecanoic acid | 0.22 | 0.20 | -0.13 | | -0.02 | **0.56**** |
| Palmitoleic (Hexadecenoic) acid 1&2 | 0.03 | 0.18 | 0.21 | | 0.08 | 0.24 |
| Palmitic (Hexadecanoic) acid | 0.13 | 0.26 | -0.24 | | -0.06 | **0.61**** |
| Methylhexadecanoic acid 1 & 2 | -0.13 | 0.27 | -0.08 | | -0.28 | -0.36 |
| Margaric (Heptadecanoic) acid | 0.22 | -0.01 | 0.04 | | 0.04 | 0.35 |
| Docosatetraenoic acid | **-0.28*** | -0.15 | -0.15 | | -0.14 | -0.18 |
| **Linoleic (Octadecadienoic) acid** | **-0.28*** | 0.19 | 0.05 | | -0.05 | **0.41*** |
| **Oleic acid & Linolenic acid** | 0.36 | 0.15 | 0.09 | | 0.10 | **0.48*** |
| Stearic (Octadecanoic) acid | **-0.42*** | -0.15 | -0.18 | | **-0.42*** | **-0.48*** |
| Octadecatrienoic acid 1 | **-0.51*** | -0.19 | -0.36 | | -0.34 | -0.27 |
| Octadecatrienoic acid 4 | -0.22 | -0.31 | 0.05 | | -0.18 | -0.34 |
| Arachidic (Eicosanoic) acid | -0.05 | -0.16 | 0.03 | | -0.06 | -0.06 |
| Behenic (Docosanoic) acid | -0.02 | -0.31 | 0.13 | | -0.05 | 0.14 |
| Nervonic (Tetracosenoic) acid | -0.06 | -0.31 | 0.11 | | -0.03 | -0.17 |
| Lignoceric (Tetracosanoic) acid 1 | 0.21 | 0.13 | -0.06 | | -0.01 | **0.41*** |
| Methyltetracosanoic acid 2 | **-0.41*** | -0.37 | -0.07 | | -0.25 | -0.35 |
| **Cerotic (Hexacosanoic) acid 2** | -0.11 | -0.01 | -0.22 | | -0.23 | **0.45*** |
| Montanic (Octacosanoic) acid | 0.30 | -0.06 | **0.45*** | | 0.26 | -0.29 |

**Table SM9:** Results of two separate (i, ii) generalized mixed effect models (GLMMs, F and ***p*-values**) analyzing the effect of (i) land-use intensity (**LUI**) and flowering plant species richness (**PSR**), and (ii) grazing (**G**), fertilization (**F**) and plant species richness (**PSR**) on single essential amino acids of *Osmia bicornis* larval pollen provisions and the 16 single fatty acids that were present in all *O. bicornis* larval pollen provisions. Note that mowing was not included in (ii) as it was significantly positively correlated with fertilization and significantly negatively correlated with grazing for our study grassland plots (see Table SM2). Year (2017 & 2018) was included as additional fixed factor and plot nested in bioregion as random factor in all models. P-values for the fixed effects included in the final most parsimonious model were calculated from F- tests based on Sattethwaite's approximation. To compare differences in variance explained by different final models we calculated R²-values (fixed effects: marginal R²: mR²; fixed and random effects: conditional R²: cR²). Plus signs indicate additive effects between fixed factors.

| **Single essential amino acids** | **Model** | ***_m_R²*** | ***_c_R²*** | ***F*** | ***p*** | **Model** | ***_m_R²*** | ***_c_R²*** | ***F*** | ***p*** |
| --- | --- | --- | --- | --- | --- | --- | --- | --- | --- | --- |
| ***Threonine*** | ***LUI*** | 0.11 | 0.15 | 4.68 | 0.06 | ***PSR*** | 0.19 | 0.46 | 3.84 | NS |
| ***Proline*** | NA | NA | NA | NA | NA | NA | NA | NA | NA | NA |
| ***Valine*** | ***LUI*** | 0.18 | 0.40 | 7.01 | < 0.05 | ***PSR*** | 0.11 | 0.63 | 3.12 | 0.09 |
| ***Methionine*** | ***LUI*** | 0.21 | 0.29 | 17.61 | < 0.001 | ***PSR*** | 0.31 | 0.42 | 6.86 | <0.01 |
| ***Isoleucine*** | ***LUI*** | 0.22 | 0.42 | 8.34 | < 0.01 | ***G*** | 0.16 | 0.58 | 8.46 | <0.001 |
| ***Leucine*** | ***LUI*** | 0.08 | 0.35 | 5.51 | NS | ***G*** | 0.19 | 0.31 | 4.20 | 0.09 |
| ***Phenylalanine*** | ***LUI*** | 0.13 | 0.45 | 7.27 | 0.056 | ***PSR*** | 0.09 | 0.46 | 2.90 | 0.06 |
| ***Lysine*** | ***LUI*** | 0.17 | 0.50 | 9.12 | < 0.01 | ***PSR*** | 0.11 | 0.57 | 3.01 | <0.001 |
| ***Histidine*** | ***LUI*** | 0.09 | 0.62 | 12.84 | NS | ***PSR***  ***+G*** | 0.20 | 0.59 | 6.78  8.08 | < 0.001  < 0.001 |
| ***Arginine*** | ***LUI*** | 0.09 | 0.41 | 7.13 | NS | ***PSR***  ***+G*** | 0.11 | 0.35 | 2.98  4.56 | 0.06  0.09 |
| **Single fatty acids** | **Model** | ***_m_R²*** | ***_c_R²*** | ***F*** | ***p*** | **Model** | ***_m_R²*** | ***_c_R²*** | ***F*** | ***p*** |
| **Caprylic (Octanoic) acid** | NA | NA | NA | NA | NA | NA | NA | NA | NA | NA |
| **Glutaric (Pentanedioic) acid** | ***LUI*** | 0.24 | 0.38 | 8.33 | <0.01 | ***PSR*** | 0.14 | 0.20 | 5.02 | <0.05 |
| **Pelargonic (Noanoic) acid** | ***LUI*** | 0.10 | 0.17 | 3.21 | NS | ***PSR*** | 0.23 | 0.32 | 6.78 | 0.09 |
| **Capric (Decanoic) acid** | ***LUI*** | 0.04 | 0.32 | 3.70 | NS | ***F*** | 0.14 | 0.53 | 4.40 | <0.001 |
| **Lauric (Dodecanoic) acid** | ***LUI*** | 0.02 | 0.10 | 1.61 | <0.01 | ***PSR*** | 0.20 | 0.48 | 4.12 | <0.001 |
| **Azelaic (Nonanedioic) acid** | ***LUI*** | 0.14 | 0.58 | 2.45 | NS | ***F*** | 0.38 | 0.81 | 5.62 | 0.057 |
| **Myristic (Tetradecanoic) acid** | ***LUI*** | 0.02 | 0.60 | 4.22 | 0.06 | ***PSR*** | 0.03 | 0.30 | 2.81 | <0.001 |
| **Palmitic (Hexadecanoic) acid** | ***LUI*** | 0.09 | 0.55 | 2.98 | 0.08 | ***PSR*** | 0.14 | 0.84 | 5.34 | <0.001 |
| **Margaric (Heptadecanoic) acid** | ***LUI*** | 0.15 | 0.73 | 5.06 | <0.05 | ***G*** | 0.30 | 0.85 | 8.32 | NS |
| **Docosatetraenoic acid** | ***LUI*** | 0.10 | 0.36 | 2.70 | <0.05 | ***F*** | 0.52 | 0.62 | 147 | NS |
| **Linoleic (Octadecadienoic) acid** | ***LUI*** | 0.04 | 0.52 | 6.62 | <0.001 | ***PSR*** | 0.16 | 0.62 | 6.86 | NS |
| **Oleic acid & Linolenic acid** | ***LUI*** | 0.01 | 0.29 | 6.50 | 0.09 | ***PSR*** | 0.04 | 0.35 | 8.07 | 0.08 |
| **Stearic (Octadecanoic) acid** | ***LUI*** | 0.12 | 0.64 | 4.23 | <0.01 | ***PSR*** | 0.28 | 0.55 | 4.88 | NS |
| **Behenic (Docosanoic) acid** | ***LUI*** | 0.06 | 0.60 | 4.12 | <0.05 | ***F*** | 0.15 | 0.78 | 4.21 | NS |
| **Lignoceric (Tetracosanoic) acid 1** | ***LUI*** | 0.01 | 0.42 | 2.02 | 0.08 | ***F*** | 0.07 | 0.62 | 4.47 | NS |
| **Cerotic (Hexacosanoic) acid 2** | ***LUI*** | 0.02 | 0.21 | 5.50 | <0.05 | ***PSR*** | 0.23 | 0.28 | 3.08 | <0.01 |

**Figures**

**Figure SM1:** Left: Artificial trap-nests for cavity nesting solitary bee species attached to the wooden fence surrounding the wheather station of each plot at the three Biodiversity Exploratories. Each trap nest contained a plastic tube with 60-80 hollow reed internodes (length ~ 20 cm, width 4-12 mm). Trap nests were checked for occupied reed internodes every 8-10 weeks from March to October to cover the whole flight period of trap-nesting solitary bees. We collected samples (i.e. occupied reed sticks) five times in 2017 and three times in 2018. Reed internodes with closed entrances were removed from plastic tubes, replaced with new reed sticks, and then directly and gently transported to the lab at the University of Würzburg (Permits for bee collections: ALB: AZ: 55-8/8848.02-07, HAI: AZ: 63.02/15.02.11-bio_expl2017.2 & AZ: 1011-17-301, SCH:AZ: 4743/128+5#69122/2018). Right: Map of the Biodiversity Exploratory framework. The Biodiversity Exploratories represent a large-scale and long-term research project investigating the impact of land-use intensity and management changes on biodiversity and ecosystem processes in three bioregions in Germany. Our study plots were geographically separated and located in the UNESCO Biosphere Reserve Swabian Alb (ALB) in southern Germany, the National Park Hainich-Dün (HAI) in central Germany and the UNESCO Biosphere Reserve Schorfheide-Chorin (SCH) in the northeast of Germany. Each of the grassland plots covered 50x50 m, yet the management type extended far beyond plot boundaries. More information on the general design of the Biodiversity Exploratories and details about plots and measurements can be found in Fischer et al. (2010).


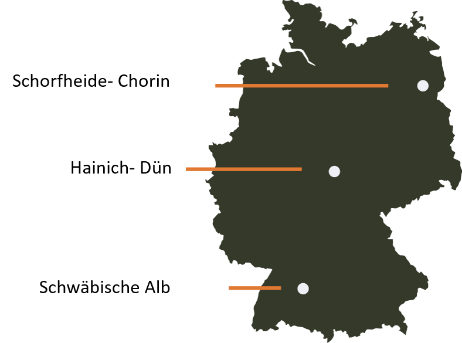


**Figure SM2:** Effects of land-use intensity (LUI) (Spearman correlations) on (A) bee species abundance, (B) bee species richness and (C) Shannon bee diversity. Different colors represent the three different bioregions (Exploratories: Schorfheide Chorin (red), Hainich-Dün (green) and Schwabian Alb (blue)) and each dot represents one plot.


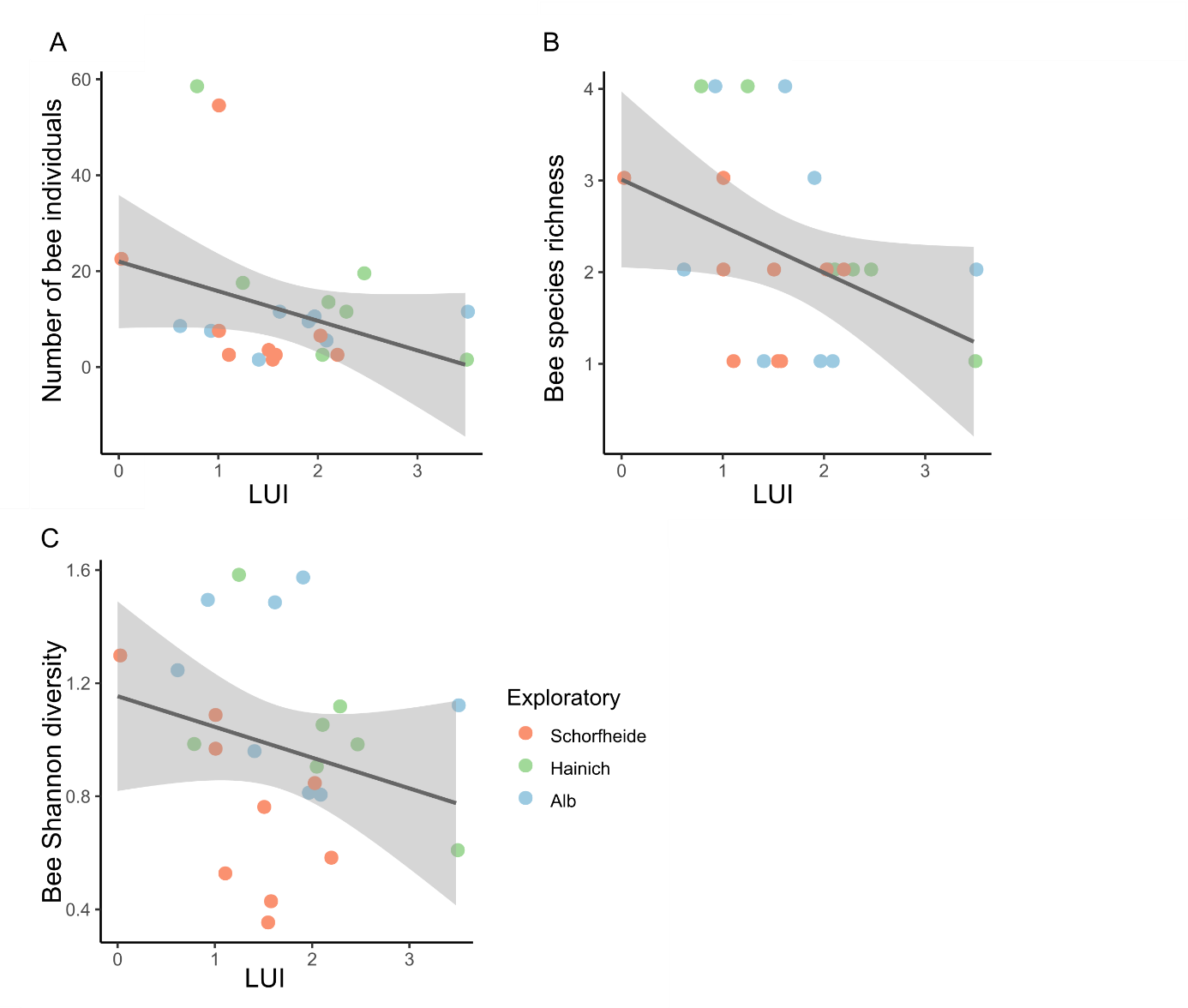


**Figure SM3:** Differences in the community composition of trap-nesting bee species found at plots of all three Exploratories (blue=Swabian Alb, green=Hainich-Dün, red=Schorfheide-Chorin) displayed by non-metrical multi-dimensional scaling (NMDS, stress = 0.16) based on the Bray-Curtis distances between bee species using their abundances (i.e. number of individuals) at each of the 27 study plots. Each dot represents one plot. Arrow obtained by environmental fitting pointing in the direction of increasing land-use intensity.


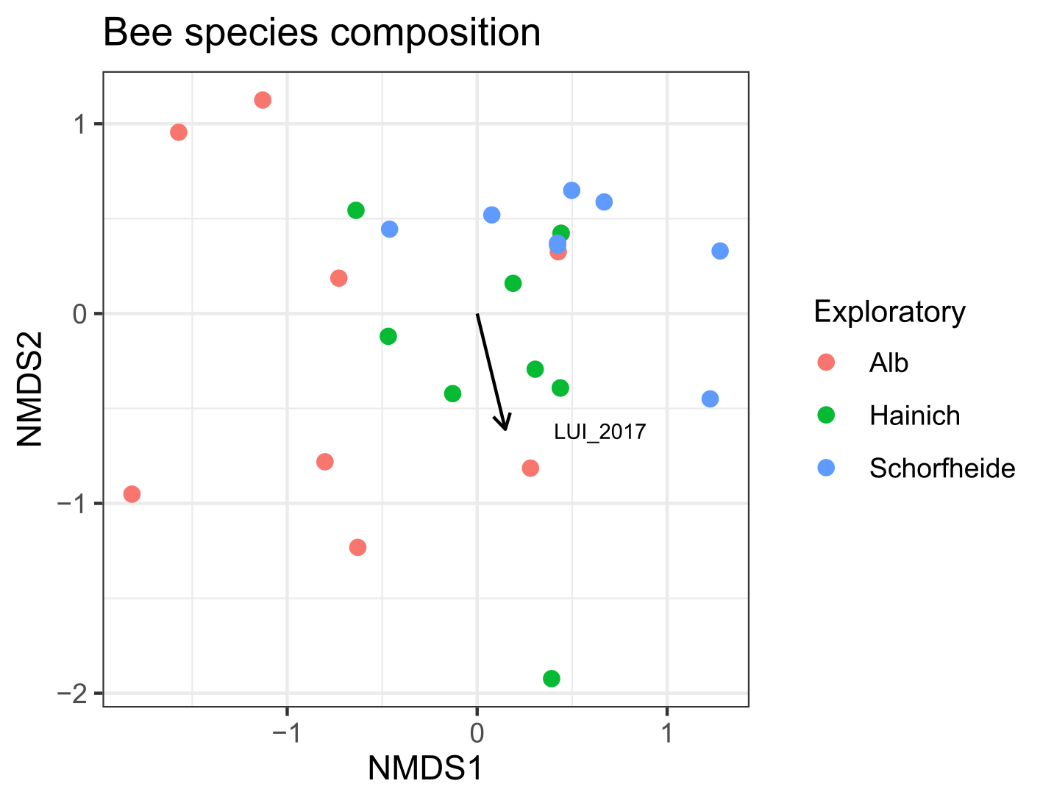


**Figure SM4:** Differences in floral taxonomic composition of larval pollen provisions between different bee species (represented by different colors) displayed by non-metrical multi-dimensional scaling (NMDS, stress=0.24) based on Bray-Curtis distances using transformed relative abundances data of ASV (amplicon sequent variants). ASVs were plotted for all bee species for all 27 plots. Each dot represents one pollen sample of one nest (*n*=150).


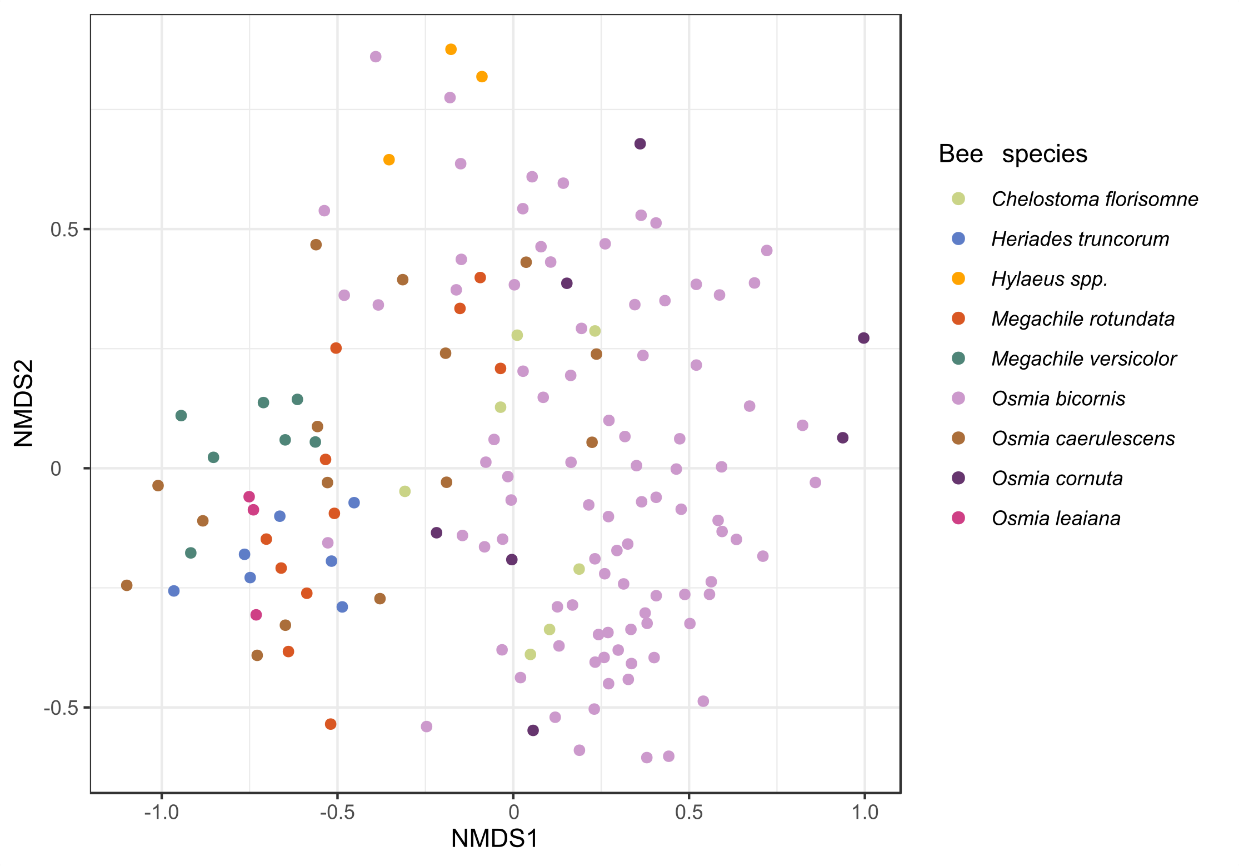


**Figure SM5:** Bipartite networks showing interactions between trap nesting solitary bee species and plant species based on larval pollen provisions sampled from nests installed at plots differing in land-use intensity and in three bioregions in Germany (Exploratories: Swabian Alb (A), Hainich-Dün (B) and Schorfheide-Chorin (C)) (assignment of ASVs up to species level). Plant species were included if they occurred in relative abundances of ≥ 1% in the respective dataset. Colored bars below bee species show occurrence of each bee species in different geographical distributions: blue: Swabian Alb, darkgreen: Hainich-Dün, red: Schorfheide-Chorin.

**
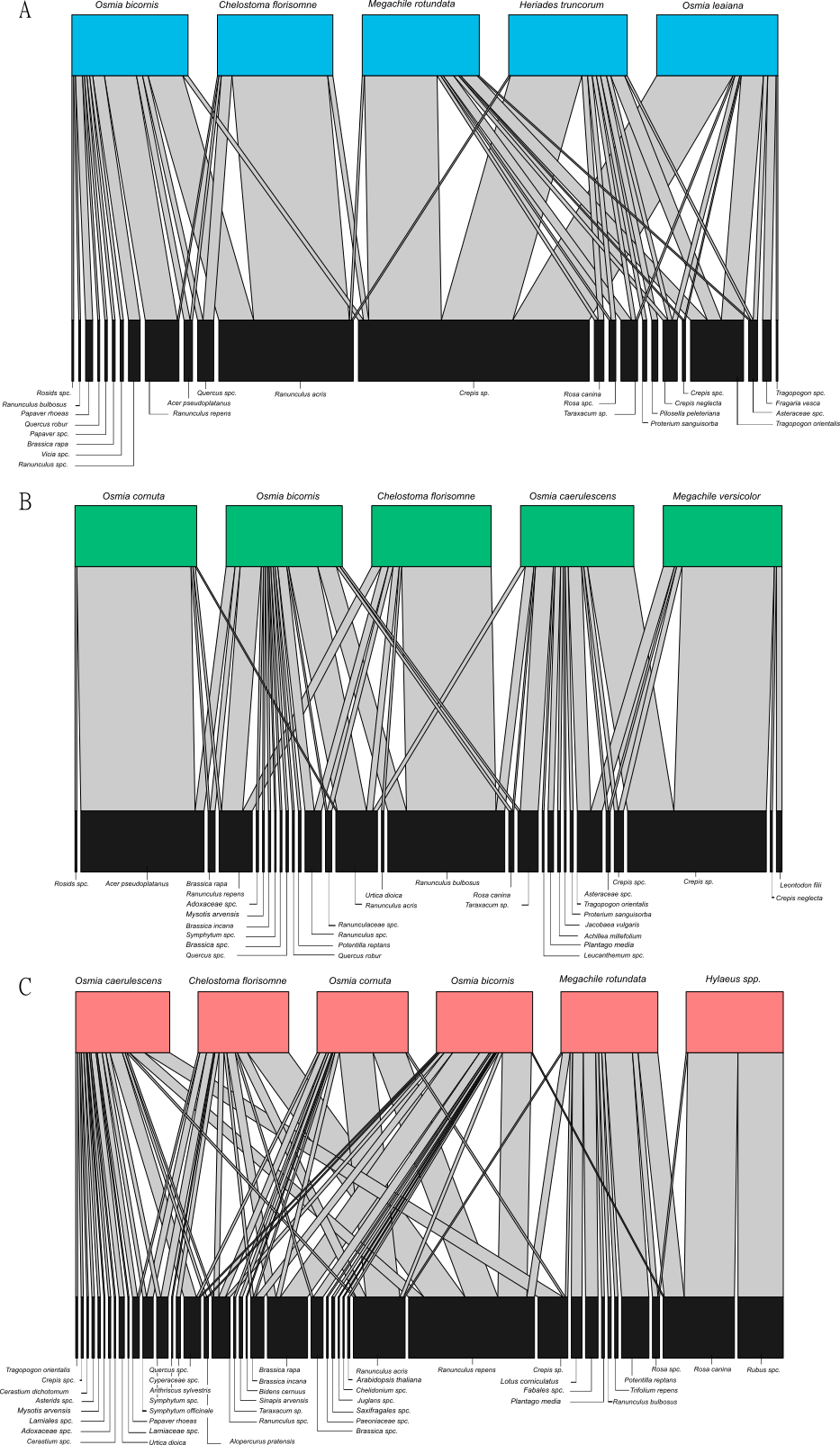
**

**Figure SM6:** Bipartite network showing plant species visited for pollen collection by *Osmia bicornis* (as found in larval provisions) at sites differing in land-use intensity (LUI); taxonomic assignments were classified at species level. All plant species occurring in relative abundances of ≥ 1% in the respective dataset were included.

**
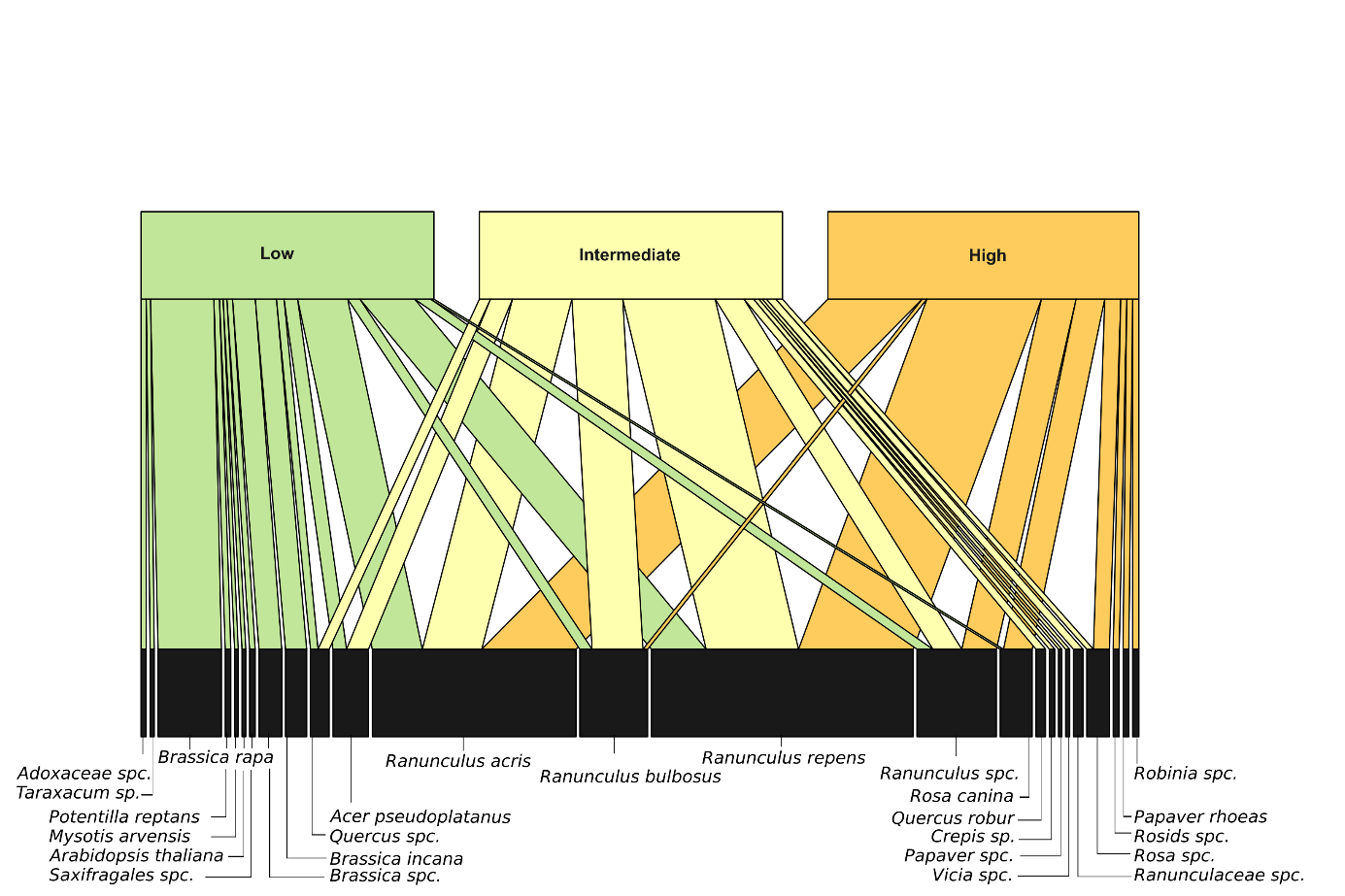
**

**Figure SM7:** Taxonomic composition of plant families in *Osmia bicornis* larval pollen provisions collected from nests located at sites differing in land-use intensity. Plant families were included if they occurred in relative abundance of ≥ 1% in the respective dataset.

**
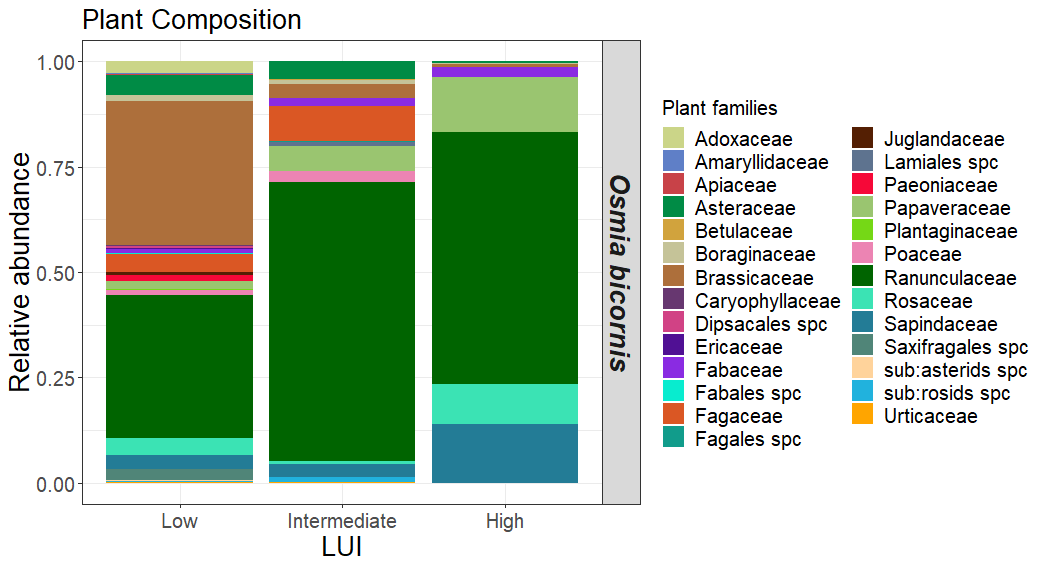
**

**Figure SM8:** Differences in pollen composition of *Osmia bicornis* larval pollen provisions collected from nests located at sites (plots) differing in land-use intensity (LUI: brown=high, orange=intermediate, lightgreen=low) displayed by non-metrical multi-dimensional scaling (NMDS, stress=0.22) based on the Bray-Curtis distances between plant species using their transformed relative abundances in each of the 90 nests collected from 27 study plots. Each point represents one *O. bicornis* pollen sample.


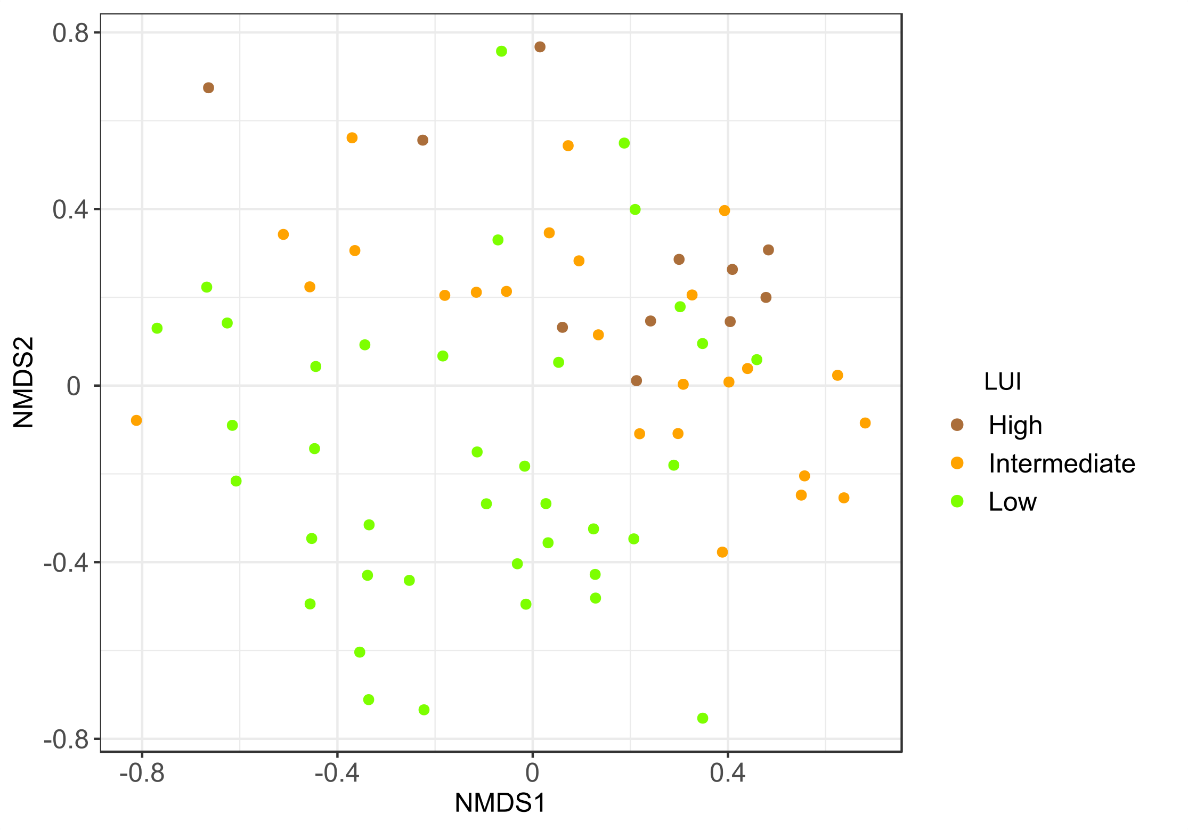


**Figure SM9:** Pearson correlation matrix between plant species and concentrations of different fatty acids found in *Osmia bicornis* larval pollen provisions from nests located at sites differing in land-use intensity at three bioregions in Germany. Colors in filled squares display correlation coefficients: color intensity correlates with correlation strength (r) and color types gives direction of correlation, i.e. positive (blue) or negative (red).

**
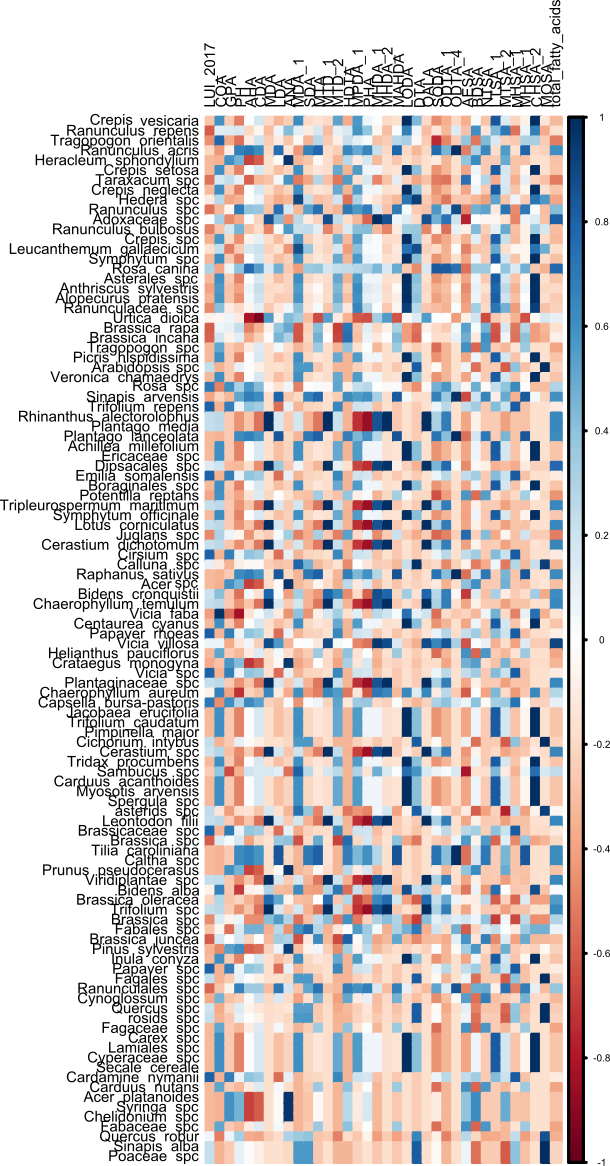
**

***References***

Ankenbrand, M., Keller, A., Wolf, M., Schultz, J., & Förster, F. (2015). ITS2 database V: Twice as much. *Molecular Biology and Evolution, 32*, 3030-3032. doi:10.1093/molbev/msv174

Edgar, R. C. (2013). UPARSE: highly accurate OTU sequences from microbial amplicon reads. *Nature Methods, 10*(10), 996-998. doi:10.1038/nmeth.2604

Edgar, R. C. (2016). SINTAX: a simple non-Bayesian taxonomy classifier for 16S and ITS sequences. *bioRxiv*, 074161. doi:10.1101/074161

Edgar, R. C., & Flyvbjerg, H. (2015). Error filtering, pair assembly and error correction for next-generation sequencing reads. *Bioinformatics, 31*(21), 3476-3482. doi:10.1093/bioinformatics/btv401

Fischer, M., Bossdorf, O., Gockel, S., Hänsel, F., Andreas, H., Hessenmöller, D., . . . Weisser, W. (2010). Implementing large-scale and long-term functional biodiversity research: The Biodiversity Exploratories. *Basic and Applied Ecology, 11*, 473-485. doi:10.1016/j.baae.2010.07.009

Keller, A., Danner, N., Grimmer, G., Ankenbrand, M., Ohe, K., Ohe, W., . . . Steffan-Dewenter, I. (2014). Evaluating multiplexed next-generation sequencing as a method in palynology for mixed pollen samples. *Plant Biology, 17*. doi:10.1111/plb.12251

Keller, A., Hohlfeld, S., Kolter, A., Schultz, J., Gemeinholzer, B., & Ankenbrand, M. (2020). BCdatabaser: on-the-fly reference database creation for (meta-)barcoding. *Bioinformatics (Oxford, England)*. doi:10.1093/bioinformatics/btz960

Sickel, W., Ankenbrand, M., Grimmer, G., Holzschuh, A., Härtel, S., & Lanzen, J. (2015). Increased efficiency in identifying mixed pollen samples by meta-barcoding with a dual-indexing approach. *BMC Ecol, 15*. doi:10.1186/s12898-015-0051-y
